# In-Vitro and In-Silico Assessment of Per- and Polyfluoroalkyl Substances (PFAS) in Aqueous Film-Forming Foam (AFFF) Binding to Human Serum Albumin

**DOI:** 10.3390/toxics9030063

**Published:** 2021-03-17

**Authors:** Wenting Li, Yuhong Hu, Heather N. Bischel

**Affiliations:** Department of Civil and Environmental Engineering, University of California Davis, Davis, CA 95616, USA; lwenting@ucdavis.edu (W.L.); 3160101554@zju.edu.cn (Y.H.)

**Keywords:** PFAS, equilibrium dialysis, bioconcentration, suspect screening, docking

## Abstract

Drinking water contaminated by fluorosurfactant-based aqueous film-forming foams (AFFF) is a source of human exposure to poly- and perfluoroalkyl substances (PFAS). However, assessment of bioaccumulation potentials of diverse PFAS in commercial products such as AFFF have been insufficient and challenging, especially due to a lack of analytical standards. Here we explore the value of suspect screening, equilibrium dialysis, and molecular-docking simulations to identify potentially bioaccumulative PFAS. We exposed human serum albumin (HSA) protein to dilutions of a legacy AFFF produced by 3M in 1999 using equilibrium dialysis and screened in-vitro protein-binding affinities using high-resolution mass spectrometry (HRMS). Through suspect screening, we identified 32 PFAS and 18 hydrocarbon surfactants in the AFFF that bound to HSA. Quantification of noncovalent association constants for 26 PFAS standards confirmed that many PFAS, including the short-chain perfluoropropane sulfonic acid (log K_a_= 4.1 ± 0.2 M^−1^), exhibit strong binding affinities with HSA. At least five PFAS in AFFF (including three PFAS with less than five perfluorocarbons) remained bound to the precipitated HSA pellet after extensive solvent washing—an indication of high PFAS binding potential. Three PFAS (PFBS, PFOS, and PFOA) were confirmed in the protein pellet with analytical standards and quantified after acid digestion—this sample fraction accounted for 5 to 20% of each compound mass in the sample. We calculated pseudo-bioconcentration factors (BCF_pseudo_) for PFAS that suspect screening flagged as noncovalently bound or potentially covalently bound. Most PFAS exhibiting high BCF_pseudo_, especially those with seven perfluorocarbons, contained a carboxylic acid or a sulfonic acid. Finally, we used molecular docking to simulate HSA binding affinities for 62 ligands (26 PFAS targets, 18 PFAS qualified in AFFF, and 18 hydrocarbon surfactants qualified in AFFF). We found that molecular docking can effectively separate HSA-binding and -nonbinding compounds in AFFF. In-vitro and in-silico approaches described in this study provide replicable, high-throughput workflows for assessing bioaccumulation potentials of diverse PFAS in commercial products.

## 1. Introduction

Application of aqueous film-forming foams (AFFF) for fire-suppression at military bases and airports is a cause of drinking water contamination with poly- and perfluoroalkyl substances (PFAS) [1]. Fluorosurfactant-based legacy AFFF formulations include complex mixtures of perfluoroalkyl carboxylic acids (PFCA), perfluoroalkyl sulfonic acids (PFSA), and highly diverse PFAS, including polyfluorinated precursors [2]. Consumption of AFFF-contaminated drinking water can lead to elevated PFAS levels in human blood [3,4]. Occupational exposure to PFAS in AFFF may also present health risks to firefighters [5,6,7]. Human exposure to PFAS has been linked to cancer, cardiovascular disease, kidney disease, liver disease, immune suppression, neurological disease, type II diabetes, osteoarthritis, respiratory disease, among other impacts [8,9].

Given these problems, researchers have gained interest in studying the health impacts of novel PFAS in AFFF [10,11], including compounds with one perfluorinated carbon that sometimes are not classified as PFAS (e.g., fluorinated aromatics) [12]. In particular, constructing physiologically-based pharmacokinetic (PBPK) models for PFAS exposure requires researchers to determine affinities (quantified as partition coefficients or association constants) among different PFAS structures in different biological tissues. The high numbers and structural diversity of existing and emerging PFAS renders this task experimentally infeasible. An alternative approach is to evaluate associations of PFAS mixtures in an AFFF with abundant model proteins (commonly serum albumin and liver fatty acid binding proteins) to identify potentially bioaccumulative PFAS and to yield quantitative relationships between PFAS exposure, bioaccumulation, and tissue distribution [13]. In this study, we assess binding affinities of diverse PFAS in AFFF to human serum albumin (HSA). HSA is the most abundant protein in human blood plasma, presenting in tissues throughout the body, and serves important biological functions (e.g., transportation of fatty acids, drugs, and thyroid hormones) [14]. Data on the binding affinities of AFFF-derived PFAS with HSA will support development of PBPK models for such PFAS.

Accurately incorporating protein binding affinities into PBPK models requires accurate understanding and quantification of the molecular mechanisms at play. Several studies reported that PFCAs and PFSAs bind with serum albumin proteins noncovalently through specific site binding or non-specific surface adsorption [15,16,17,18]. Two studies identified a potential for covalent binding between PFAS and albumin proteins [19,20]. While no studies have investigated ultrastrong noncovalent bindings (K_A_ > 10^9^ M^−1^) between PFAS and proteins, ultrastrong binding was observed for PFAS in aqueous supramolecular polymerization [21]. Common serum-extraction protocols are likely to overlook or discard strongly bound ligands, including covalently bound or ultrastrong noncovalently bound PFAS. In organic solvent extraction, for instance, the precipitated protein pellet is disposed after extraction—along with any strongly bound ligands or residual targets. In online or offline solid phase extraction (SPE), covalently bound ligands and denatured proteins are lost on SPE cartridges [22]. Additionally, many studies accounted for matrix effects by spiking calibration standards into blank serum before analysis [23]. Extraction efficiencies are typically reported as satisfactory (e.g., 70–130% spike recoveries) by using such matrix (serum) matched calibration curves for quantification, thereby inadvertently masking strong protein–ligand interactions [24,25].

**In this study, we combined experimental and modeling techniques to identify potentially bioaccumulative PFAS present in an AFFF and to investigate multiple binding pathways for diverse PFAS structures.** We first utilized HSA as a model protein system to quantify both noncovalently bound and potentially covalently bound PFAS using targeted analysis. This analytical approach facilitates evaluation of the degree to which strongly bound or residual PFAS may be discarded in precipitated protein pellets. We then estimated bioconcentration factors for legacy and novel PFAS using linear (L)-PFOS as a bioaccumulative benchmarking compound. Finally, we predicted protein binding affinities for novel PFAS using molecular docking, a traditional drug design tool that simulates interactions between small molecules and large proteins [26,27]. The results comprised the most comprehensive quantification of relative PFAS-HSA binding affinities to date, providing valuable inputs for bioaccumulation and PBPK models.

***PFAS Terminology***

In this paper, we adopted acronyms and rules established in previous studies for PFAS terminology. We mainly followed acronym naming rules established by Barzen-Hanson et al. (2017) [2]. We followed the rules published by Buck et al. (2011) for naming polyfluoroalkyl phosphate esters (PAPs) and fluorotelomer acrylates [28]. Table S6.2 details our abbreviation rules for compounds that were not previously characterized with abbreviated names. The number of perfluorocarbons (n) in a compound is indicated as C_n_, which should not be confused with the number of carbons in PFAS. For instance, PFOA is a C_7_ PFCA, while PFOS is a C_8_ PFSA. Based on categorizations from the Organisation for Economic Co-operation and Development (OECD) [12], we used the term “short-chain” to refer to PFCAs containing fewer than six perfluorocarbons and PFSAs with fewer than five perfluorocarbons. We used the term “long-chain” to refer to PFCAs containing six or more perfluorocarbons and to PFSAs with five or more perfluorocarbons. We applied the short-chain and long-chain convention for PFCAs to all other PFAS evaluated in this study. For analogs of well-known PFSAs identified, we added common contractions to enhance recognizability. For instance, PFOS analogs include chloro (Cl), ketone (K), unsaturated (U), hydrogen (H), linear (L), and branched (br)-substituted PFOS [29].

## 2. Materials and Methods

### 2.1. Study Design and Workflow

The overall workflow consists of the following components. First, we exposed HSA to either (a) dilutions of an AFFF produced by 3M in 1999 or (b) in-house mixtures of 26 PFAS (listed in Table S6.2) through equilibrium dialysis. We used data-dependent (tMS/MS, for target PFAS) and data-independent (all-ion fragmentation, for suspect-screening) mass spectrometry against an in-house library of PFAS and hydrocarbon surfactants to identify compounds that were bound noncovalently to HSA. Second, we acid-digested residual protein pellets that were free of noncovalently bound PFAS. We applied suspect screening to identify residual PFAS in the precipitated protein pellet. Residual PFAS were considered candidates for forming ultrastrong or covalent associations with HSA. We used targeted MS for three PFAS to quantify residual levels in the protein pellet. Third, we quantitatively evaluated protein association constants predicted by molecular docking between 26 target PFAS structures and two HSA crystal structures. Fourth and finally, we used the molecular docking workflow to classify PFAS and non-PFAS surfactants in the AFFF as either HSA-binding or non-binding.

### 2.2. Equilibrium Dialysis

Equilibrium dialysis was performed in a 96-well system (Harvard Apparatus, Holliston, MA, USA) in which each polypropylene cell was separated into two chambers by a 10-kDa regenerated cellulose membrane. One side of each dialysis cell was dosed with 7.97 mg HSA (≤0.02% fatty acids, Sigma-Aldrich, Munich, Germany) in phosphate-buffered saline (PBS, pH 7.4 prepared in HPLC grade water) to a final concentration of 600 μM, which mimics physiological conditions [30]. The other side of each cell was then dosed with 200 μL of an AFFF dilution (4000 to 16,000-fold in PBS) or an in-house mixture of 26 PFAS (prepared with PFAC-24PAR, PFPrS, and br-PFOS from Wellington Laboratories Inc., Guelph, ON, Canada). Seventeen out of the 26 target PFAS in this study are commonly measured in drinking water using EPA method 533 or EPA method 537.1.

An aliquot of legacy AFFF (3M, 1999) was provided by Professor Christopher Higgins at the Colorado School of Mines. Experimental batches consisted of six concentration levels of AFFF or PFAS standard dilutions and were replicated four times. For each batch of experiments, a method blank was prepared with HSA free of AFFF or PFAS. A negative control cell containing AFFF or the PFAS standard mix was prepared without HSA to assess free movement of PFAS through the membrane (Figure S1.1). The system was incubated at 37 °C while rotating at 30 RPM for 108 h (Figure S1.2).

### 2.3. PFAS Extractions

**Figure 1:**
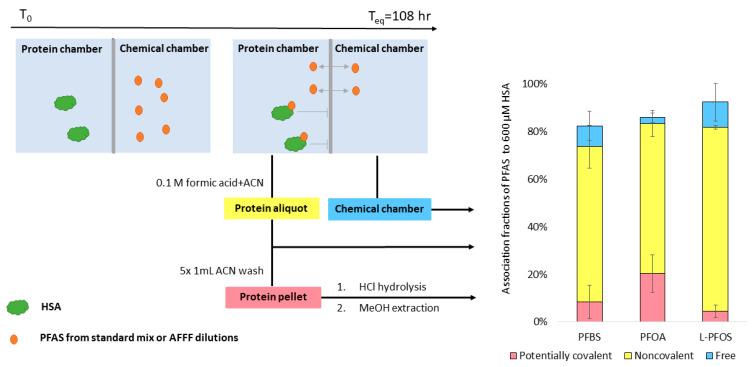
Equilibrium dialysis set-up and observed mass balance for three PFAS with initial dosages of 40–80 ng. PFAS that were free in aqueous solution PFAS, noncovalently bound PFAS, and residual PFAS in the precipitated protein pellet were measured independently. The time required to reach equilibrium (T_eq_) was previously determined using 26 PFAS standards in Figure S1.2. Detailed mass balance data are available in Table S1.1.

Aliquots from each dialysis cell were processed to generate three extracts, shown in Figure 1. These were (1) free PFAS from the aqueous fraction, (2) noncovalently bound PFAS associated with the dissolved protein, and (3) residual PFAS in the precipitated protein pellet (candidates for ultrastrong noncovalently bound or potentially covalently bound PFAS). Briefly, post-dialysis aqueous samples (100 μL) from the chemical chambers were equilibrated with 50% methanol. Protein aliquots (100 μL) of the post-dialysis protein cells were extracted with formic acid (FA) acidified acetonitrile (ACN) for protein denaturation and precipitation. Noncovalently bound PFAS were determined by taking the concentration difference between protein aliquot fraction and chemical fraction. The residual protein pellets were further washed with 1 mL ACN five times, and the last wash was concentrated (to 200 μL) and saved to verify the absence of PFAS. We then applied a standard acid hydrolysis protocol for amino acid analysis to break peptide bonds and to release any PFAS that were possibly covalently bound to HSA [31,32,33,34,35] Extracts of noncovalently bound PFAS and residual PFAS in the protein pellet were solvent exchanged into 50% methanol to match the final solvent composition of the aqueous extracts. Additional details on the sample preparation can be found in Supporting Information (S1.1). An internal standard mix (ISTD, Table S6.1) in 50% methanol was added into each extract prior to analysis. Details on instrumentation and acquisition settings are provided in Table S5.1.

### 2.4. Analytical Instrumental Set-Up

Quantification of 26 PFAS targets and qualification of diverse PFAS via suspect screening were performed on each AFFF dilution and each PFAS extract from dialysis. Data were acquired on an Agilent 1260 Infinity HPLC system paired with a 6530 QTOF MS. Sample extracts (10 μL) were injected onto a C18 column (ZORBAX RRHD Eclipse Plus C18 column; 2.1 mm × 150 mm, 1.8 µm, Agilent Technologies, Inc., Santa Clara, CA, USA) at a flow rate of 0.40 mL/min, with a total run time of 31.5 min. The aqueous mobile phase (A) was 20 mM ammonium acetate (Fisher Scientific, Pittsburgh, PA, USA) in Optima^TM^ HPLC grade water (Fisher Scientific, Pittsburgh, PA, USA) and the organic mobile phase (B) was 100% Optima^TM^ HPLC grade acetonitrile (Fisher Scientific, Pittsburgh, PA, USA). The mass spectrometer ionized samples in a negative mode using collision energies (CE) of 0, 10, 20, and 40 eV. A quality-control run of the 26-PFAS standard mix was analyzed after every 8 samples to ensure that concentrations of targeted PFAS remained within 30% of known concentrations.

### 2.5. Suspect Screening

For suspect screening, mass-to-charge-ratios (*m/z*) of 50–1200 were fragmented in the collision cell with CE of 0, 10, 20, and 40 eV in All-Ions acquisition modes. Data were processed using Agilent MassHunter Qualitative Analysis (B.08.00) by applying the “Find by Formula” search against an in-house AFFF Personal Compound Data Library (PCDL). The PCDL contained 3793 PFAS extracted from the Norman Suspect List Exchange (OECDPFAS) [36], and 727 hydrocarbon surfactants (monoisotopic mass: 100–1200) extracted from the surfactant suspect list curated by Schymanski et al. (2014) [37]. The PCDL also included 63 MS/MS spectra, of which 31 spectra were acquired from in-house standards, 24 spectra were extracted from MassBank [38], and six spectra were generated with CFM-ID 3.0 [39]. PFAS were considered qualified with level 2–3 confidence as outlined in Schymanski et al. (2014) [40]. Suspect-screening search settings are listed in Table S5.2. Qualified PFAS were reported only if the following additional criteria were met: (1) the abundance of the qualified ion was greater than three times the experimental blank ion abundance; (2) the ion was qualified in at least 67% of dialysis cells in each batch of experiments; and (3) the PFAS qualified in dialysis cell extracts were also qualified in neat AFFF dilutions. All qualified PFAS that met these additional criteria were confirmed by re-running extracts with data-dependent targeted analysis (see Section 2.6).

### 2.6. Targeted MS/MS

For data-dependent targeted analysis, a list of targeted mass-to-charge ratios (*m/z*) and corresponding retention times (RT) was compiled based on pre-runs with All-Ions acquisition described in Section 2.5. To avoid overlapping peaks of targeted compounds, duplicate injections were performed for each sample to ensure at least 0.4 min RT difference between peaks of any two targeted compounds within one injection. This approach can minimize false identifications of PFAS due to the instrument’s inherent mass error.

### 2.7. PFAS Quantification

Quantification was performed with 19-ISTD dosed ten-point calibration curve (0.5–250 ng/mL). Whole method limits of quantification (LOQ) range from 0.025 to 1 ng/mL. Details of analytical standards and extraction recoveries are available in Table S6.1.

### 2.8. Experimental Determination of PFAS Noncovalent Binding Affinities

The concentrations of 26 PFAS targets that partitioned into the protein chamber, remained in the chemical chamber, and remained associated with the protein pellet were directly determined by HPLC-QTOF-MS. Noncovalent binding affinities, measured as association constants (K_A_), were calculated assuming a one site specific binding as shown in Equations (1) and (2).

(1) $$\left[\mathrm{HSA}\right]+\left[{\mathrm{PFAS}}_{i}\right]\overset{k_{\mathrm{on}}}{\underset{k_{\mathrm{off}}}{⇌}}\left[{\mathrm{HSA}-\mathrm{PFAS}}_{i}\right]$$

(2) $$\frac{\left[{\mathrm{HSA}-\mathrm{PFAS}}_{i}\right]}{\left[\mathrm{HSA}\right]\left[{\mathrm{PFAS}}_{i}\right]}=\frac{k_{on,i}}{k_{off,i}}=K_{A,\;i}$$

To enable the assessment of multiple specific binding sites, we also fitted the data to the Langmuir isotherm model [41] with a limited binding sites assumption, following Equations (3) and (4).

(3) $$q_{m}=\left[{\mathrm{HSA}-\mathrm{PFAS}}_{i}\right]+q_{0,i}$$

(4) $$\frac{1}{\left[{\mathrm{HSA}-\mathrm{PFAS}}_{i}\right]}=\frac{\left[{\mathrm{PFAS}}_{i}\right]*q_{0,i}}{\frac{1}{K_{A,\;i}}+\left[{\mathrm{PFAS}}_{i}\right]}$$

In these equations, ***i*** refers to the compound of interest, ***q_m_*** is the concentration of total binding sites, and ***q*****_0,*i*_** is the concentration of empty binding sites. $\left[{\mathrm{PFAS}}_{i}\right]$ is the concentration of free PFAS***_i_*** measured in the chemical side of the equilibrium dialysis set-up. $\left[{\mathrm{HSA}-\mathrm{PFAS}}_{i}\right]$ is calculated by taking the difference between the concentration of PFAS in the protein side and in the chemical side, as shown in Figure 1. Additional isotherm models, including linear adsorption and Freundlich adsorption models, were also evaluated (Figure S1.3).

### 2.9. Computational Simulations of Noncovalent PFAS Protein Binding

We used AutoDock Vina (v 1.1.2) [42] to dock 62 ligands (26 PFAS targets, 18 qualified PFAS, and 18 qualified hydrocarbon surfactants in AFFF) to two HSA crystal structures (Protein Data Bank entries 1E7G and 1AO6). 1E7G was chosen as the native structure of HSA, which complexed with tetradecanoic acid (myristic acid) [43,44]. 1AO6 is an unliganded HSA structure and may be more similar in conformation to the HSA we used experimentally, since the protein standard we purchased contained low levels of fatty acids (<0.02%). We followed the workflow outlined by Ng and Hungerbuehler (2015) with several modifications. Specifically, in the ligand-preparation step, we used the “Generating Conformers” function in DataWarrior V5.2.1 [45] to generate 3D structures for all ligands. Then, we optimized ligand structures using the MMFF94s forcefield in Avogadro V1.90.0 [46]. In addition, we used PyMol (v2.3.3) [47,48] for structure visualization, redocking alignment, and crucial residue identification. Simulations were repeated 100 times for 6 binding pockets, and each simulation generated 9 binding modes, yielding 5400 predictions in total for each PFAS. Further details on the docking method as well as simulation precision and accuracy are available in Supporting Information (S3.1 and S3.2).

The simulation method was evaluated by redocking PFOS on an experimentally determined HSA (Protein Data Bank entry 4E99) structure that was originally complexed with two PFOS in fatty-acid binding site (FA) 3/4 and 5. The atomic root-mean-square-deviation (RMSD) of redocked PFOS on FA 3/4 and FA 5 was determined to be less than two angstroms, indicating successful redocking. The redocking search information and RMSD statistics are available in Table S2.3.

## 3. Results

### 3.1. Characterization of PFAS in AFFF

Targeted analysis using 26 PFAS standards was insufficient for characterizing the AFFF sample: less than 9% of the total organic fluorine was quantified as compared to quantitative ^19^F NMR (Table S7.1). In addition to the target PFAS, we identified 18 other PFAS and 18 hydrocarbon surfactant structures using suspect screening analysis for initial qualification. The suspected PFAS and hydrocarbon surfactant structures were further confirmed via data-dependent acquisition or library spectrum match. Manual annotation of the MS/MS spectra supported identification of these compounds (S1.2, Figures S4.1–S4.19). Based on structural categorizations conducted by the OECD [12], PFAS qualified in the AFFF sample included: 19 perfluoroalkane sulfonyl compounds, seven perfluoroalkyl carbonyl compounds, four fluorotelomer-related compounds, and two side-chain fluorinated aromatic compounds. Eight PFAS suspects were qualified in the initial screening but eliminated via manual confirmation. A full list of the hydrocarbon surfactants identified in AFFF is provided in Table S6.2. These hydrocarbon surfactants homologous series were detected using EnviHomolog (http://www.envihomolog.eawag.ch accessed on 5 November 2020). A repeating mass-increment of 14.0156 (-CH_2_-) was observed for four Linear Alkylbenzyl Sulfonates (LAS). Repeating mass-increments of 28.0313 (-C_2_H_4_-) and 44.0262 (-C_2_H_4_O-) were observed for 31 Alkyl Ethoxy Sulfates (AES).

### 3.2. Noncovalent and Potentially Covalent Binding of PFAS in AFFF to Human Serum Albumin

Of 32 PFAS identified in the AFFF, 28 PFAS bound noncovalently to HSA in equilibrium dialysis experiments. We confirmed 14 of these PFAS with analytical standards, and the remaining via data-dependent acquisition. Five PFAS were qualified and confirmed in the precipitated and washed protein pellets. Since PFAS released from hydrolyzed HSA pellets could not be extracted with the organic solvent, natural dissociation of this fraction of PFAS was not expected in a reasonable timeframe. Hence, these PFAS were considered as candidates for ultrastrong noncovalent or potentially covalent binding to HSA. Fourteen additional PFAS were qualified (confidence level 4) in the protein pellet, but were not qualified in the AFFF dilutions. We excluded these compounds from further analysis.

In HSA binding experiments using the 26 PFAS targets, three PFAS (PFBS, PFOA, and PFOS) were consistently quantified in the protein pellets. The protein pellets contained 7% PFBS, 20% PFOA, and 5% PFOS of the total spiked mass (80 ng) of each of these compounds (Figure 1). PFHxS was detected inconsistently in the protein pellets (11 out of 24 samples among four trials). The AFFF (df = 4 × 10^3^)-spiked protein pellets contained 1% of spiked PFBS, 26% of spiked PFOA, and 2% of spiked PFOS. N-(3-(dimethylamino)propyl)-1,1,2,2,3,3,4,4,5,5,6,6,6-tridecafluorohexane-1-sulfonamide FHxSA (N-diMAmP-FHxSA) and 4,4,4-trifluoro-2-(2,2,2-trifluoroethyl)butanoic acid (diTF-IsoBA) were also detected consistently in the protein pellets (at least 16 out of 18 pellets among 3 trials) after AFFF exposure, but could not be quantified due to lack of available standards.

### 3.3. Quantitative Determination of PFAS–HSA Association Constants

**Figure 2:**
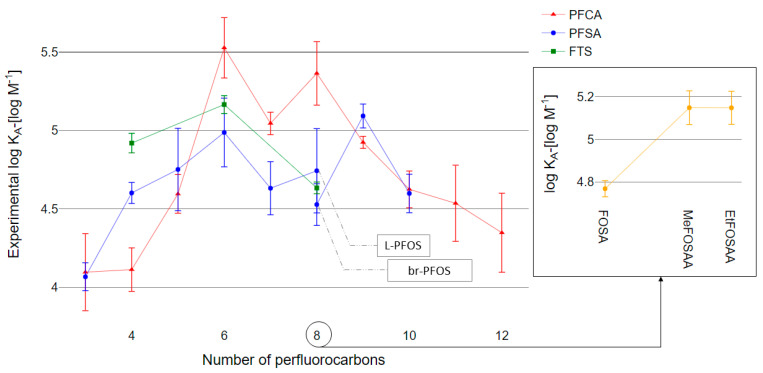
Experimentally determined noncovalent association constants (K_A_) of 26 PFAS targets with human serum albumin (has) in equilibrium dialysis. Three C_8_ precursor compounds, Perfluoro-1-octanesulfonamide (FOSA), N-methylperfluoro-1-octanesulfonamidoacetic acid (MeFOSAA), and N-ethylperfluoro-1-octanesulfonamidoacetic acid (EtFOSAA) are presented in a separate plot. The error bars represent one standard deviation.

We experimentally quantified HSA binding affinities for 26 PFAS: 11 PFCAs (C_3_ through C_13_), nine PFSAs (C_3_ through C_12_), one perfluoroalkane sulfonamide (C_8_), two perfluoroalkane sulfonamide acetic acids (methylated and ethylated C_8_), and three fluorotelomer sulfonic acids (4:2 FTS, 6:2 FTS, and 8:2 FTS). PFAS-HSA association constants ranged from 10^4.0^ to 10^5.5^ M^−1^ (Figure 2).

### 3.4. Evaluation of Molecular Docking to Predict the PFAS–HSA Binding Affinities

**Figure 3:**
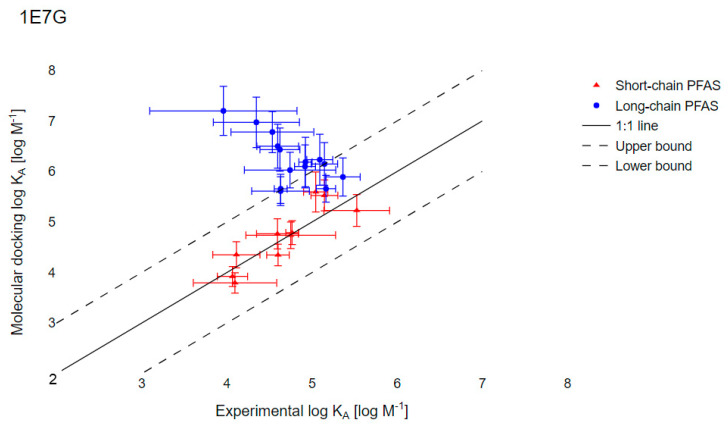
Comparison of experimental log K_A_ with results from molecular simulations with the HSA crystal structure 1E7G. Solid black lines represent the 1:1 line; dotted lines represent one log unit higher or lower. Error bars reflect one geometric standard deviation (GSD).

Molecular docking of PFAS with two HSA crystal structures (1E7G and 1AO6) was used to simulate K_A_ for the 26 PFAS tested experimentally (Table S7.2). Accurate K_A_ predictions using 1E7G were limited to short-chain PFAS. In Figure 3, for the nine short-chain PFAS (PFCA with less than six perfluorinated carbons and PFSA with less than five perfluorinated carbons), a significant positive correlation between the docking-predicted K_A_ and the experimentally determined values was observed (95% CI: slope = 1.02 ± 0.19, r = 0.900). For the 17 long-chain PFAS, significant negative correlation between the docking-predicted K_A_ and the experimentally determined values was observed (95% CI: slope = −1.05 ± 0.30, r = 0.7680).

## 4. Discussion

### 4.1. AFFF Formulation

Most PFAS identified in this study were also reported by Houtz et al. (2013) [49], Barzen-Hanson et al. (2017) [2], and McDonough et al. (2020) [29] for the same or similar AFFF commercial products. We qualified C_4_ and C_6_ perfluoroalkyl sulfonamide amino carboxylates and perfluoroalkyl sulfonamido amines that were reported by Houtz et al. One pentafluorosulfide-containing eight perfluorocarbon PFAS (8-F5S-PFOS) found in AFFF was also reported by Barzen-Hanson et al. We qualified four of six PFOS-substituted compounds (H-PFOS, U-PFOS, Cl-PFOS, and K-PFOS) and one of two PFDS-substituted compounds (H-PFDS) reported by McDonough et al. A C_6_ containing phosphonic acid and ester functional groups (8:2 monoPAP-diEes) identified in this study was detected in PFAS-contaminated soil (from paper sludge) in Germany [50]. We identified six novel PFAS that have not been otherwise detected in environmetnal samples to our knowledge: 4-FHp-CycHxA, diTF-IsoBA, Hx-diFB, Uridine-FB, and two C_4_ fluoroalkyl esters (N-PFBS-MFPe, N-FBEAc). Fluoroalkyl esters may undergo hydrolysis in the ambient environment and eventually release PFSAs or PFCAs [51].

### 4.2. HSA Noncovalent Binding Affinity Relative to Perfluorocarbon Chain Length

Consistent with previous studies of PFAS-protein associations, PFAS were highly bound to HSA [24,25,41]. HSA contains multiple PFAS binding sites with potentially different binding affinities [18,52] such that measured K_A_ values represent a mixture of affinities for different binding sites. A majority of the PFAS exhibited linear binding isotherms, indicating nonspecific noncovalent associations with HSA (Figure S1.4 and Table S1.1). K_A_ followed an inverted-V trend by which K_A_ increased with perfluorocarbon chain elongation up to C_6_ through C_9_ and subsequently decreased (Figure 2). The trend for C_4_ through C_6_ and C_8_ through C_11_ PFCAs is consistent with the pattern for bovine serum albumin (BSA)–water distribution coefficients (K_PW_) determined by Bischel et al. (2011) [25]. The PFSA trend for C_4_ through C_8_ is consistent with the BSA-association constants determined by Allendorf et al. (2019) [24]. Our measurements of K_A_ were generally an order of magnitude lower than K_A_ measured by Allendorf et al. (2019), with the exception of PFBA, PFHpA, and C_6_ PFAS. We have greater confidence in the physiological relevance of our experimental results as our results were obtained at physiologically relevant molar ratios of PFAS and HSA, and our K_A_ values were determined from isotherm data rather than single-point experimentation. As low levels of AFFF exposures to humans are most common [53], we tested PFAS-HSA association constants from 0.001 to 0.1 PFAS:HSA.

Overall, a trend of increasing K_A_ with perfluorocarbon-chain length was observed for PFCAs and PFSAs up to C_6_. The HSA binding affinities of perfluorohexanesulfonic acid (PFHxS) and perfluoroheptanoic acid (PFHpA) were exceptionally high (Log K_A_: 4.99 ± 0.44 and 5.53 ± 0.39, respectively). These observations are consistent with long blood plasma elimination half-lives reported for PFHxS in humans [54,55] and for PFHpA and PFHxS in pigs [56].

### 4.3. Residual PFAS in Precipitated Protein Pellets

**Figure 4:**
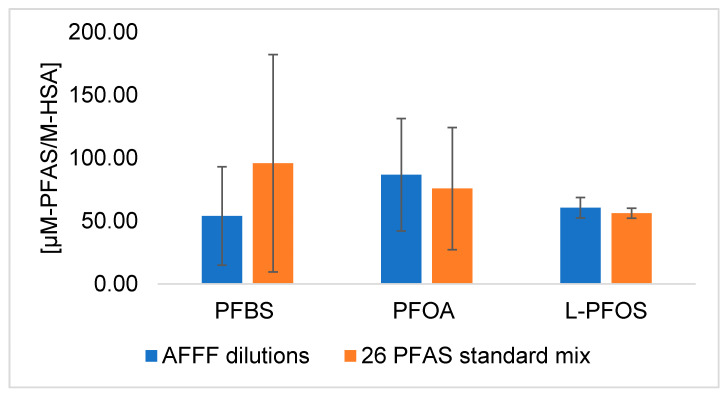
Residual PFAS quantified in digested HSA pellets, precipitated from equilibrium dialysis experiment.

Consistent detection of three PFAS in the digested protein pellets precipated from AFFF and PFAS standard exposure experiments is presented in Figure 4. Residual protein pellets from HSA exposed to PFAS standards (which did not include C_4_ precursors) contained similar amount of PFBS, PFOA, and PFOS as protein pellets from HSA exposed to AFFF dilutions (Figure 4 and Table S1.1). PFAS release from the protein pellet could be a result of several factors. First, residual PFAS in the pellet could be present as an analytical artifact resulting from high-concentrations of PFAS in AFFF. However, no quantifiable level of PFAS was observed in the protein pellet washes, blank cells, or control cells used in the equilibrium dialysis experiment (See Figure S1.1). Additionally, AFFF was diluted from 2000-fold to 80,000-fold prior to HSA exposure in the most dilute case, and the PFAS targets were still present in the precipitated protein from these tests. Second, PFAS could be retained in the pellet via ultrastrong noncovalently interations, which was observed in an amphiphilic polymerization system [21]. In conjunction with an exterior aqueous environment, large proteins like HSA that have multiple hydrophobic binding sites can provide an amphiphilic environment in which different protein residues interact with the polar headgroups and hydrophobic perfluorotails of PFAS simultaneously [57]. Third, PFAS could be retained in the pellet via covalent interations, the potential for which we evaluate in further detail below.

To our knowledge, only two types of PFAS have been reported to bind covalently to proteins: PFCAs [19] and fluorotelomer unsaturated aldehydes (FTUALs) [20,58]. The mechanism of covalent binding between PFAS and proteins remains unclear, but thiol- and nitrogen-containing nucleophilic amino acids in serum albumin proteins are suspected to play a role [19]. In the case of FTUALs, covalent bond formation occurs via Michael addition [58]. This mechanism cannot explain our observation of perfluoro alkyl carboxylic and sulfonic acids in the protein pellet. Formation of covalent bonds between carboxylic acids or sulfonic acids containing ligands and protein residues has not been observed under physiological conditions [59,60]. The low mole ratio of residual PFAS detected in the protein pellet to initial HSA levels indicates that not all PFAS-HSA associations resulted in PFAS retention in the protein pellets.

While we are unable to disentangle the mechanisms explaining PFAS in the protein pellet, we consider residual PFAS in protein pellets as candidates for ultrastrong noncovalent or potentially covalent binding to HSA. In addition to the perfluoro alkyl acids (PFAAs) described above, we noted that 14 additional PFAS qualified in the dialysis extracts were not further evaluated in this study (i.e., by acquiring targeted MS/MS). It is possible that these PFAS were generated from reactions with HSA or through transformations during the acid-hydrolysis processing step. We would expect the formation of PFAS-HSA covalent bonds and subsequent digestion of PFAS-containing HSA to yield PFAS-peptide complexes. Future analysis should evaluate whether perfluorocarbon moieties are associated with amino acids or peptides following digestion.

### 4.4. Semi-quantification of Bioconcentration Factors of Qualified PFAS

**Figure 5:**
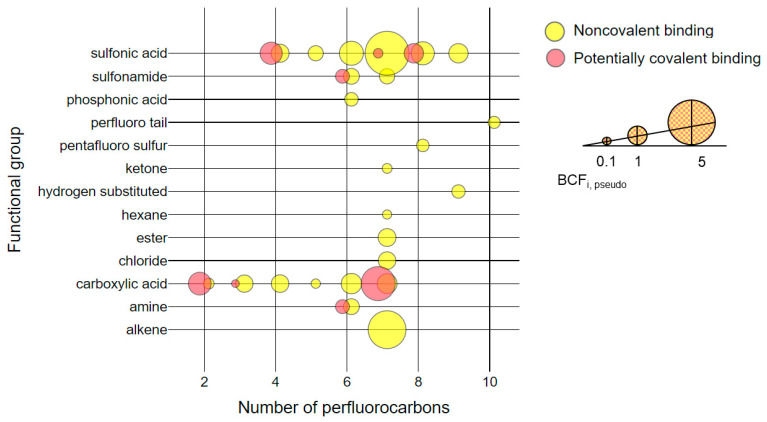
Pseudo-bioconcentration factors (BCF_i,pseudo_ ) of PFAS in aqueous film-forming foams (AFFF) that were bound noncovalently (yellow) or potentially covalently (red) to HSA. Bubble size represents the natural logarithm of the BCF_pseudo_ (legend BCF_pseudo_ = 1). For 12 qualified PFAS with multiple functional groups, a separate bubble of the same size is displayed for each functional group (e.g., noncovalently bound Cl-PFOS contained chloride and sulfonic acid groups and is represented as two bubbles with seven perfluorocarbons).

We calculated pseudo-bioconcentration factors (BCF_pseudos_) for noncovalently bound and potentially covalently bound fractions separately to evaluate patterns related to perfluorocarbon chain length and functional groups (Figure 5). BCF_pseudo_ serves as a quantitative benchmarking technique to cross-compare bioaccumulation potentials of novel PFAS using qualitative screening data [29]. The BCF_pseudo_ was calculated for each PFAS as follows:

(5) $${\mathrm{BCF}}_{i,pseudo}=\frac{A_{i,\mathrm{sample}}}{A_{L-\mathrm{PFOS},\mathrm{sample}}}*\frac{A_{\text{L-PFOS,AFFF}}}{A_{i,\mathrm{AFFF}}}$$

where ***A_i_***_,sample_ is the peak area of compound *i* detected in the protein aliquot or pellet; ***A***_L-PFOS,sample_ is the peak area of L-PFOS detected in the protein aliquot or pellet; ***A_L-PFOS,AFFF_*** is the peak area of L-PFOS detected in neat AFFF dilutions; and ***A_i_***_,AFFF_ is the peak area of compound ***i*** detected in neat AFFF dilutions. All peak areas were normalized with their corresponding internal standard peak area prior to the calculations.

Analysis of BCF_pseudo_ revealed several key findings in Figure 4. First, sulfonic acids (C_4_ through C_9_) and carboxylic acids (C_2_ through C_7_) consistently exhibited high BCF_pseudos_ in the noncovalently bound fraction. Second, C_7_ PFAS across different functional groups consistently exhibited high BCF_pseudos_ in the noncovalently bound fraction. We observed a noncovalent binding trend with a turning point at C_7_ for all qualified PFAS, which is consistent with observations for targeted PFSAs (Figure 2). Third, PFOA exhibited the highest BCF_pseudo_ for potentially covalently bound fractions. This is despite low levels in the AFFF; PFOA represented less than two percent of the total organic fluorine mass in the AFFF among all PFAS compounds quantified through targeted analysis. Finally, three PFAS with four or fewer perfluorocarbons exhibited higher BCF_pseudos_ in the potentially covalently bound fraction than L-PFOS. The BCF_pseudo_ for the C_2_ carboxylic acid in the potentially covalently bound fraction (BCF_diTF-IsoBA, pseudo_ = 13.8 ± 3.14) was an order of magnitude greater than the BCF_pseudo_ for L-PFOS. This finding is concerning, as short-chain PFAS are typically considered less bioaccumulative than long-chain PFAS and exhibit weaker noncovalent interactions with proteins [61]. Our results indicate that short-chain PFAS may in fact be strongly retained in proteins (and in precipitated protein pellets) even when present at low concentrations in serum. Further studies should consider the impacts of these observations on analytical conclusions as well as potential toxicological risks.

To assess the ability of in-vitro binding studies with HSA to represent bioaccumulation potentials of PFAS in animals, we compared our BCF_pseudos_ from the noncovalently bound fraction to pseudo-bioaccumulation factors (BAF_pseudos_) calculated in an in-vivo mouse-dosing study that used the same AFFF commercial product [29]. Our calculation of BCF_pseudo_. (Equation 5) was equivalent to the calculation of BAF_pseudo_ by McDonough et al. (2020). However, we performed direct exposure of HSA to PFAS while McDonough et al. used a PFAS sample from mouse serum following AFFF oral gavage. Twelve types of PFAS (43 distinct chemical structures) were qualified by McDonough et al. in the mouse serum (which excluded analysis of the protein pellet and associated ligands) following oral gavage of the AFFF. Of the six reported noncovalent BAF_pseudos_ for PFOS substitutes prevalent in mouse serum, two were similar to the noncovalent BCF_pseudos_ we observed noncovalently bound to HSA. The bioconcentration potential of U-PFOS with seven perfluorocarbons was high in both studies (BCF_U-PFOS,pseudo_ = 8.85 ± 0.16 with HSA, compared to BAF_U-PFOS,pseudo_ = 6.7 in mouse serum, averaged across genders). The bioconcentration potential of noncovalently associated Cl-PFOS (BCF_Cl-PFOS,pseudo_ = 0.82 ± 0.16 for HSA) was similar to L-PFOS (BCF_L-PFOS,pseudo_ = 1) in both McDonough et al. and this study. Comparisons between the two studies may otherwise aid in identifying products of metabolic biotransformation. For example, the mouse serum BAF_pseudo_ for hydrogen-substituted PFAS (H-PFOS) in McDonough et al. was about one order of magnitude higher than the BCF_H-PFOS,pseudo_ we observed for HSA. We suspect that biotransformation—which only takes place for in-vivo experiments—might have contributed to the high BAF_H-PFOS,pseudo_ observed by McDonough et al., given that H-PFOS is a daughter product of precursor PFAS in AFFF. Altogether, these results indicated the value of comparing BAF_pseudo_ and BCF_pseudo_ to assess contributions of protein binding and metabolism of PFAS precursors as explanatory factors for PFAS bioaccumulation.

### 4.5. Molecular Docking Predictions

Molecular docking simulations provide a rapid and high-throughput strategy to assess protein–ligand interactions. However, the accuracy of AutoDock Vina for the best protein–ligand conformations is only 60 to 80% [62]. Modeling assumptions integrated into AutoDock Vina include, for example, unrealistic rigidity of the protein and the removal of water [63]. The docking scores generated and shown in Figure 3 and Figure S2.3 (for 1AO6) therefore cannot be used directly as protein–ligand binding energies, especially for large compounds. Nevertheless, we expected that docking predictions for short-chain PFAS would be more accurate than for long-chain PFAS, as docking accuracy declines with increasing numbers of rotatable bonds [42]. Indeed, we observed a positive correlation for short-chain PFAS between docking scores generated and our experimental results (Figure 3), while docking scores for long-chain PFAS were negatively correlated with experimental results.

To the best of our knowledge, Ng and Hungerbuehler (2015) [27] conducted the only other PFAS–HSA molecular docking study, predicting binding affinities of 25 PFAS with HSA. As shown in Figure S2.4, the two studies were in good agreement (least-square-linear correlation predicted K_A_ for 1E7G slope = 0.993, r = 0.997), even though our mean docking scores were calculated based on 5400 conformations (available in Table S7.2) instead of the 54 conformations simulated by Ng and Hungerbuehler. Unlike the previous study, we did not apply a 20-fold correction factor to decrease the predicted K_A_ values, as this did not improve correlations with our experimentally determined K_A_ values. Ng and Hungerbuehler may have required a correction factor due to the diversity of experimental results compiled in their study and unrealistic conditions in available studies. Many studies evaluated PFAS-HSA binding affinities using oversaturated protein conditions. For instance, Chen and Guo (2009) measured the HSA association constants of PFAS at ligand:protein ratio ranging from 4 to 40 in their fluorescence quenching study [64]. Han et al. (2003) used microdesalting columns to measure the PFAS–HSA association constants with a ligand:protein ratio ranging from 4 to 36 [65]. When the ligand-to-protein ratio is greater than one, PFAS can shift from primary binding site(s) to secondary binding sites [41]. PFAS associations with secondary binding sites on HSA are not physiologically relevant, as human exposure to PFAS generally occurs at low levels [66]. Our experimental data represent binding affinities at primary binding site(s) because we made direct measurements with high-resolution-mass-spectrometry at low ligand:protein mole ratios.

### 4.6. Qualitative Prediction of HSA-bound vs. Nonbound Compounds

**Figure 6:**
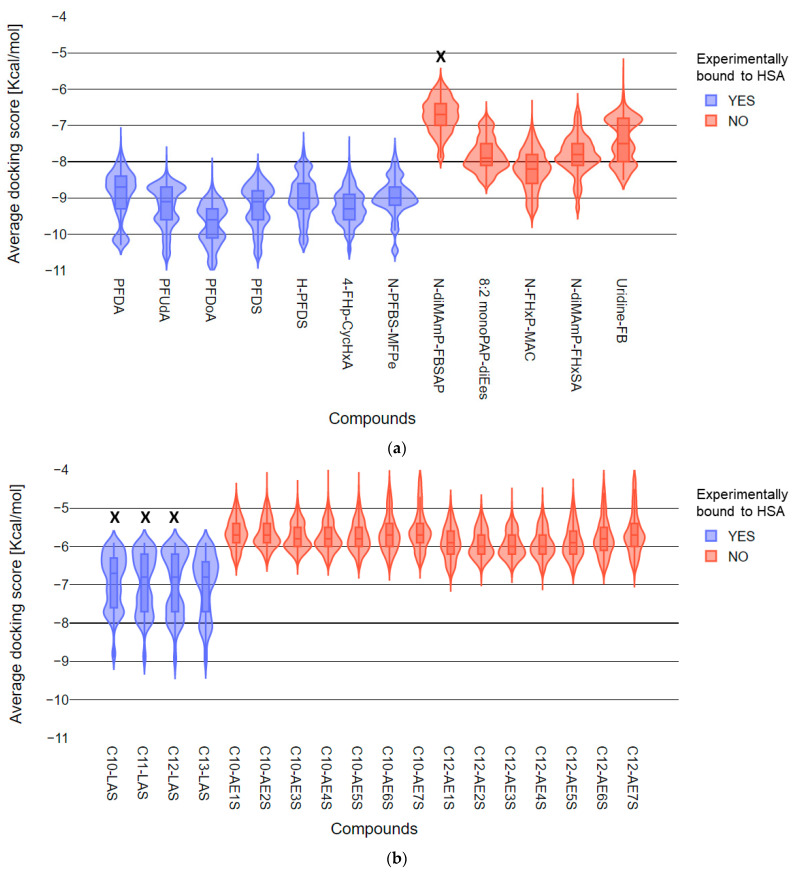
Violin plots of molecular docking simulated 1E7G docking scores for (**a**) PFAS with greater than 10 perfluorocarbons and (**b**) hydrocarbon surfactants identified in AFFF that have greater than 10 carbons. The shape of each violin represents a rotated kernel density plot of 5400 HSA–PFAS binding conformations generated from simulations for six binding pockets. Blue and red colors are used to distinguish experimental results. The compounds with significantly greater peak area (after correction with ISTD peak area) in the noncovalently bound fraction of the protein chamber relative to the chemical chamber are shown in blue. The compounds identified experimentally in the protein chamber that were not significantly greater in peak area relative to the chemical chamber are shown were red. Distributions in red were significantly different than distributions in blue Kruskal–Wallis (*p* < 0.05) except for those distributions marked with a black X.

While docking scores are unreliable for quantitative predictions of binding affinities, docking scores can be used for qualitative comparisons between complexes and to identify candidate ligands for the protein of interest. We used AutoDock Vina to identify HSA-binding compounds in AFFF by comparing PFAS and hydrocarbon surfactants in AFFF. Results of docking are presented as violin plots, which display the distribution of simulated docking scores (Figures S3.1–S3.6). For each PFAS, a kernel density plot of docking scores was derived using the 5400 conformations generated from simulations on six HSA binding pockets. All hydrocarbon surfactants identified in AFFF had more than 10 carbons in the backbone, so we selected docking results for PFAS with 10 or more perfluorocarbons in the backbone for comparison (Figure 6). For both sets of long-chain PFAS and hydrocarbon surfactants, we observed a separation of distributions that appeared to distinguish binding (low docking scores) or non-binding (high docking scores) compounds. The low and high docking scores corresponded, respectively, to PFAS that were observed as bound or unbound to HSA in in-vitro experiments. We performed a Kruskal–Wallis test of docking predicted binding scores for the compounds in Figure 6. Most compounds in AFFF that we observed to bind to HSA in the in-vitro experiments also had predicted docking scores that were significantly different from the unbound compounds (*p* < 0.05 in Table S7.3). However, the binding score of one experimentally determined unbound PFAS (N-diMAmP-PBSAP) and three experimentally determined bound hydrocarbon surfactants (C10-LAS, C11-LAS, and C12-LAS) were not significantly different from each other (*p* = 1, in Table S7.3, labeled with black X in Figure 6). Experimental results for suspect screening were thus largely consistent with simulated docking scores when comparing HSA binding affinities within the same class of chemicals.

The shape of the kernel density plots may also provide insights into different binding processes. The kernel density plots for both bound and unbound PFAS (Figure 6a and Figures S2.5–S2.7) are more varied in shape than those for hydrocarbon surfactants (Figure 5b and Figure S3.0). Similar to kernel plots, cluster analyses were commonly used to identify preferential ligand binding sites [67], suggesting site-specific binding between PFAS and HSA [68]. Predicted HSA binding scores for bound hydrocarbons exhibited bimodal or even trimodal (C10-LAS and C11-LAS) distributions. Predicted HSA binding scores for nonbound hydrocarbons converged and centralized for all HSA binding pockets (Figures S2.8 and S2.9). To further validate and accurately predict PFAS-HSA binding energies, mechanistic studies using molecular dynamics coupled with molecular mechanics/Poisson–Boltzmann surface area (MM/PBSA) methods for these PFAS are ongoing in our research group.

## 5. Conclusions

Our study explored the value of suspect screening and computational simulations to identify potentially bioaccumulative PFAS from a PFAS-containing commercial product, AFFF. A majority of the PFAS we identified in a legacy AFFF bind to the most abundant protein in human serum, human serum albumin (HSA). At least five PFAS, including two PFAS with less than five perfluorocarbons, were detected in the precipitated and washed protein pellet. The potential health implications of ultrastrong or covalent binding of PFAS are unclear. Covalent modifications of HSA affect the clearance and metabolic destiny of many drugs, and have been hypothesized as the center of toxicity exhibited by many drugs [69,70,71].

Our observation of binding of short-chain PFAS to albumin is concerning and requires further mechanistic assessment. Short-chain PFAS are largely considered less bioaccumulative, with shorter half-lives in organisms, than long-chain PFAS. Our results indicated that some short-chain PFAS may be retained in the blood for much longer—these PFAS remained associated with HSA after extensive solvent washing. Computational simulations for bioaccumulation potential can provide value by decreasing reliance on time- and labor-intensive laboratory experiments. Though predicted binding scores cannot quantitively describe binding affinities with HSA, the scores can be used to qualitatively identify previously uncharacterized PFAS that are likely to bind to HSA. More broadly, this study offers a framework for evaluating bioaccumulation potentials of thousands of PFAS in comparable biological tissues.

## Data Availability

All data generated or analyzed during this study are included in this published article (and its supplementary files).

## Supplementary Materials

The following are available online at https://www.mdpi.com/2305-6304/9/3/63/s1, S1.1: Details on the serum extractions, S1.2 Manual annotation of PFAS MS/MS spectra, S2.1: Materials used in 19F NMR, S2.2: Details on 19F qNMR method, S2.3: Details on 19F qNMR data acquisition method, S3.1: Details on molecular docking, S3.2: Details on ligand preparation, Figure S1.1: Experimental setup for equilibrium dialysis, Figure S1.2: Assessment of equilibration time required for equilibrium dialysis, Figure S1.3: Isotherm models for PFAS adsorbed to HSA, Figure S1.4: Bound fractions of PFAS compounds in AFFF(df = 1 × 10^4^) to HSA, Figure S2.1: Crystal structures of Human Serum Albumin (HSA), Figure S2.2: Predicted binding scores of 26-PFAS binding to HSA (1E7G), Figure S2.3: Molecular docking predicted association constants (KA ) of 26 PFAS to HSA, Figure S2.4: Uncorrected KA values for 1E7G from this study correlate to the results reported by Ng and Hungerbuehler (2015), Figure S3.1: Histogram of molecular docking predicted target PFCA-1E7G binding scores, Figure S3.2: Histogram of molecular docking predicted target PFSA-1E7G binding scores, Figure S3.3: Histogram of molecular docking predicted target PFAS(precursor)-1E7G binding scores, Figure S3.4: Histograms of molecular docking predicted scores for C10-AEnS binding 1E7G, Figure S3.5: Histograms of molecular docking predicted scores for C12-AEnS binding 1E7G, Figure S3.6: Histograms of molecular docking predicted scores for Cn-LAS binding 1E7G, Figure S4.0: Homologous plot of hydrocarbon surfactants in AFFF and HSA aliquots (RT vs. *m/z*), Figure S4.(1–19): Annotated MS/MS spectrum of qualified compounds in ESI-, Table S1.1: Mass (ng) of PFAS present in each fraction of equilibrium dialysis extracts, Table S2.1: Molecular docking searching information for 1E7G, Table S2.2: Molecular docking searching information for 1AO6, Table S2.3: Molecular docking searching information for 4E99, Table S5.1: LC-QTOF-MS acquisition method (LC-QTOF-MS acquisition), Table S5.2: Suspect screening software search algorithm criteria for LC-QTOF-MS acquired data (Screening parameters), Table S5.3: 19F and 1H NMR acquisition method (NMR acquisition), Table S6.1: List of LC-QTOF-MS targeted compounds, internal standards, LOQ, and extraction recoveries (LCMS target compounds), Table S6.2: Qualified PFAS in AFFF using suspect-screening (LCMS qualified PFAS), Table S6.3: Qualified hydrocarbon surfactants in AFFF using suspect-screening (LCMS qualified nonPFAS), Table S7.1: AFFF formulation comparison of total fluorine quantified in AFFF using HPLC-QTOF-MS and 19F NMR (Total fluorine quant), Table S7.2: Experimentally determined and molecular docking predicted HSA-PFAS association constants (Ka_docking and exp), Table S7.3: Kruskall-Wallis one-way pairwise ANOVA analysis on docking predicted HSA (1E7G) binding affinities of long-chain PFAS and hydrocarbon surfactants identified in AFFF (Kruskall-Wallis ANOVA).
