# Correction: Li et al. In-Vitro and In-Silico Assessment of Per- and Polyfluoroalkyl Substances (PFAS) in Aqueous Film-Forming Foam (AFFF) Binding to Human Serum Albumin. *Toxics* 2021, *9*, 63

**DOI:** 10.3390/toxics10060297

**Published:** 2022-05-31

**Authors:** Wenting Li, Yuhong Hu, Heather N. Bischel

**Affiliations:** Department of Civil and Environmental Engineering, University of California Davis, Davis, CA 95616, USA; lwenting@ucdavis.edu (W.L.); 3160101554@zju.edu.cn (Y.H.)

## Text Correction

The authors wish to make the following corrections to this paper [1]:

There was an error in the original publication. In Section 4.2, the article stated, “HSA binding affinities of perfluorohexanesulfonic acid (PFHxS) and perfluoroheptanoic acid (PFHpA) were exceptionally high (Log K_A_: 5.89 ± 0.55 and 5.74 ± 0.38, respectively)”. The Log K_A_ values were inconsistent with Figure 2 and the SI7.2.

The corrected paragraph is as follows.

“The HSA binding affinities of perfluorohexanesulfonic acid (PFHxS) and perfluoroheptanoic acid (PFHpA) were exceptionally high (Log K_A_: 4.99 ± 0.44 and 5.53 ± 0.39, respectively)”.

The authors apologize for any inconvenience caused and state that the scientific conclusions are unaffected. The original publication has also been updated.
