# Supplementary material for: In-Vitro and In-Silico Assessment of Per- and Polyfluoroalkyl Substances (PFAS) in Aqueous Film-Forming Foam (AFFF) Binding to Human Serum Albumin
Source: Toxics. 2021 Mar 17;9(3):63. doi: 10.3390/toxics9030063 (PMC8002870; doi:10.3390/toxics9030063)
Supplement: Supplementary file 1 [file toxics-09-00063-s001.zip › toxics-1134374-SI.pdf]

toxics MDPI

Article

Supporting Information: Qualitative and quantitative assessment of legacy and novel per- and polyfluoroalkyl substances (PFAS) in Aqueous Film-Forming Foam (AFFF) binding to human serum albumin

Wenting Li [1], Yuhong Hu [1] and Heather N. Bischel *

Content

S1.1 Details on the serum extractions

S1.2 Manual annotation of PFAS MS/MS spectra

S2.1 Materials used in 19F NMR

S2.2 Details on 19F qNMR method

S2.4 Details on 19F qNMR data acquisition method

S3.1 Details on molecular docking

S3.2 Details on ligand preparation

Figures

Figure S1.1 Experimental setup for equilibrium dialysis

Figure S1.2 Assessment of equilibration time required for equilibrium dialysis

Figure S1.3 Isotherm models for PFAS adsorbed to HSA

Figure S1.4 Bound fractions of PFAS compounds in AFFF(df=1E4) to HSA

Figure S2.1 Crystal structures of Human Serum Albumin (HSA)

Figure S2.2 Predicted binding scores of 26-PFAS binding to HSA (1E7G)

Figure S2.3 Molecular docking predicted association constants (KA) of 26 PFAS to HSA

Figure S2.4 Uncorrected KA values for 1E7G from this study correlate to the results reported by Ng and Hungerbuehler (2015)

Figure S3.1 Histogram of molecular docking predicted target PFCA-1E7G binding scores

Figure S3.2 Histogram of molecular docking predicted target PFSA-1E7G binding scores

Citation: Li, W.; Hu, Y.; Bischel, H.N. In-Vitro and In-Silico Assessment of Per- and Polyfluoroalkyl Substances (PFAS) in Aqueous Film-Forming Foam (AFFF) Binding to Human Serum Albumin. *Toxics* **2021**, *9*, 63. https://doi.org/10.3390/toxics9030063

Received: 18 February 2021
Accepted: 14 March 2021
Published: 17 March 2021

Publisher's Note: MDPI stays neutral with regard to jurisdictional claims in published maps and institutional affiliations.

Copyright: © 2021 by the authors. Licensee MDPI, Basel, Switzerland. This article is an open access article distributed under the terms and conditions of the Creative Commons Attribution (CC BY) license (http://creativecommons.org/licenses/by/4.0/).

Figure S3.3 Histogram of molecular docking predicted target PFAS(precursor)-1E7G binding scores

Figure S3.4 Histograms of molecular docking predicted scores for C10-AEnS binding 1E7G

Figure S3.5 Histograms of molecular docking predicted scores for C12-AEnS binding 1E7G

Figure S3.6 Histograms of molecular docking predicted scores for Cn-LAS binding 1E7G

Figure S4.0 Homologous plot of hydrocarbon surfactants in AFFF and HSA aliquots (RT vs m/z)

Figure S4.(1-19) Annotated MS/MS spectrum of qualified compounds in ESI-

Tables

Table S1.1 Mass (ng) of PFAS present in each fraction of equilibrium dialysis extracts

Table S2.1 Molecular docking searching information for 1E7G

Table S2.2 Molecular docking searching information for 1AO6

Table S2.3 Molecular docking searching information for 4E99

Excel File

Tables S5 to S7 are located in a separate excel file (Li2021_toxics_SupportingInformation_Tables.xls).

Table S5.1 LC-QTOF-MS acquisition method (LC-QTOF-MS acquisition)

Table S5.2 Suspect screening software search algorithm criteria for LC-QTOF-MS acquired data (Screening parameters)

Table S5.3 19F and 1H NMR acquisition method (NMR acquisition)

Table S6.1 List of LC-QTOF-MS targeted compounds, internal standards, LOQ, and extraction recoveries (LCMS target compounds)

Table S6.2 Qualified PFAS in AFFF using suspect-screening (LCMS qualified PFAS)

Table S6.3 Qualified hydrocarbon surfactants in AFFF using suspect-screening (LCMS qualified nonPFAS)

Table S7.1 AFFF formulation comparison of total fluorine quantified in AFFF using HPLC-QTOF-MS and 19F NMR (Total fluorine quant)

Table S7.2 Experimentally determined and molecular docking predicted HSA-PFAS association constants (Ka_docking and exp)

Table S7.3 Kruskall-Wallis one-way pairwise ANOVA analysis on docking predicted HSA (1E7G) binding affinities of long-chain PFAS and hydrocarbon surfactants identified in AFFF (Kruskall-Wallis ANOVA)

S1.1 Details on serum extractions

A schematic of a dialysis cell experiment setup is shown in Figure S1.1. After the system reached equilibrium, 100 uL of solution from the chemical side was transferred along with 25 ng internal standard (ISTD) mix and 100 uL of methanol (MeOH) for HPLC-QTOF-MS analysis. Then, 100 uL of HSA solution was added into an Eppendorf tube with 200 uL of 0.1 M formic acid and 1.7 mL cold (-20 °C) acetonitrile (ACN) for protein denaturation and precipitation. The sample tube was vortexed and centrifuged (16,800 ⊚g) for 3 min to separate the protein pellet and the aliquot. The aliquot fractions were solvent exchanged into MeOH, concentrated to 200 µL, and equilibrated with 25 ng of ISTD mix. To assess if any ultrastrong or covalent bonds were present, loosely associated PFAS in the HSA pellet were washed with 2 mL of ACN five times, and the last fraction was saved to confirm PFAS levels were below detection following the approach adopted by Vanden Heuvel et al. (1992) [1]. After evaporating residual ACN, dry protein pellets were hydrolyzed with 50 µL of 6 N HCl in closed cap Eppendorf tubes at 110 °C for 24 h [2–5]. Excess HCl was evaporated under N2, and the hydrolyzed pellet was neutralized with NaOH and solvent exchanged to MeOH. After syringe filtration (Captiva Agilent Premium Syringe Filter, 2 µm, regenerated cellulose membrane), protein pellet extracts were equilibrated with internal standards and ready for analysis. Details on the native and internal standards used are given in the Supporting Information Excel file under tab S6.1 LCMS target compounds.

S1.2 Manual annotation of PFAS MS/MS spectra

For MS/MS spectra annotation, we used an in-silico fragmentation tool and literature reported common ESI- fragments. We acquired data-dependent MS/MS spectra of suspect-screening qualified compounds using retention time and tentative molecular m/z from the original runs and then used MetFrag Web Tool (https://msbi.ipb-halle.de/MetFragBeta/, accessed on 11/05/2020) to identify fragmented ions, following the parameter settings reported by Moschet et al. (2018) [6]. Furthermore, we manually screened the fragment ion list extracted from MS/MS spectra against the "Fragments ESI-" published as supporting information by Barzen-Hanson et al. (2017) [7]. In this manual annotation step, we assigned Schymanski's [8] confidence level 3 to the compounds with at least one fragment ion in addition to the presence of molecular ion(s). We assigned the confidence level 2b to the compounds with at least 4 fragmented ions that could piece together the molecular ion. In addition to [M-H], the [M-CH3COO] adduct was used as qualifier due to abundant acetate ions from the aqueous mobile phase. Example MS/MS spectra annotations are presented in Figure S4.1 to S4.20.

S2.1 Materials used in 19F NMR

The following materials were used in 19F NMR analysis of AFFF: (1) solvents: methanol-d4 (MeOD) 99.96%D (Sigma Aldrich: 444758); deuterium oxide-d2 99.9%D (Sigma Aldrich: 151882); HEPES buffer (Sigma Aldrich: 83264); (2) chemical shift reference compound: fluorotrichloromethane (CFCl3), 99+%, (Sigma Aldrich: 25499-1); (3) calibrating standard addition compounds: 2,2,2-trifluoroacetamide (TFAcAm), 97%, (Sigma Aldrich: 14465-7); 2,2,2-trifluoroethanol (TFE), 99%, (Polysciences, Inc:612197); (4) target compounds: perfluorooctanoic acid (PFOA), 95%, (Sigma Aldrich: 171468); perfluorooctanesulfonic acid (PFOS), 100 ug/mL, (Supelco: 33607); perfluoro-2-propoxypropanoic acid (PFPrOPrA, "GenX"), 97%, (Synquest Laboratories: 2121-3-13); 1H,1H,2H,2H-Perfluorodecan-1-ol, 99%, (Synquest Laboratories: 2101-3-95); perfluorobutanesulfonic acid

(PFBS), 97%, (Synquest Laboratories: 6164-3-09); perfluoro butanoic acid (PFBA), 98%, (Synquest Laboratories: 2121-3-34); perfluoroglutaric acid, 97%, (Sigma Aldrich: 196908).

S2.2 19F-NMR method

19F and 1H NMR spectroscopy were performed with a 500-MHz Bruker Avance DRX NMR spectrometer equipped with a quad probe with 90º power pulse (acquisition settings shown in Table S5.3). The total fluorine quantification method was developed based on a report by the United States Naval Research Laboratory with some modifications [9]. The chemical shift of the sample was calibrated against fluorotrichloromethane (CFCl3) in methanol-d4 (CD3OD) and deuterium oxide-d2 (D2O) at 0 ppm. For total fluorine quantification, trifluoroethanol (TFE) was used as the internal standard in a solvent mixture of CD3OD and methanol.

Two internal calibration standards (TFE and TFAcAm) were tested, and they were both calibrated with CFCl3 (δF= 0 ppm). When no additional PFAS standard was added, TFE/CD3OD (δF= -77.08 +/-0.10 ppm) appeared to have more consistent chemical shift compared to TFAcAm/CD3OD (δF= -75.78 +/-0.49 ppm). Moreover, as the model compound (e.g., PFOA) was added into the NMR tube, a decrease in intensity for TFAcAm was observed. Therefore, total fluorine quantification was performed solely with TFE. For total fluorine quantification in AFFF, a constant volume of 10% TFE (20 μL) in MeOD was added into NMR quartz tubes (Wilmad: 16-800-338) along with 90 μL of AFFF or calibration standard dilutions, 450 μL MeOD, and 1 mL of CFCl3 vapor. As shown in Equation (1), the concentration of total fluorine content in AFFF can be determined with fluorine signal ratios of TFE with known fluorine content and AFFF dilutions with varied dilution factors.

$$\frac{\boldsymbol{C_{TFE(F)}\,V_{TFE}}}{\boldsymbol{C_{AFFF(F)}V_{AFFF}}} = \frac{\boldsymbol{A_{TFE}}}{\boldsymbol{A_{AFFF}}} \qquad \boldsymbol{Equation\ (1)}$$

In this equation, C_(TFE(F)) is the fluorine concentration in the TFE dilution (mg/μL), V_TFE is the volume of the TFE dilution (μL), C_(AFFF(F)) is the fluorine concentration in the AFFF dilution (mg/μL), V_AFFF is the volume of the AFFF dilution (μL), A_TFE is integrated peak area of 19F resonance caused by TFE, and A_AFFF is the sum of integrated area of 19F resonance caused by PFAS in AFFF. We tested the accuracy of the method with GenX dilutions, achieving a percent error of 3.8±3% for triplicated samples (R^2>0.9995).

S3.1 Details on molecular docking

We used AutoDock Vina (v1.1.2) to simulate PFAS–protein interactions. AutoDock Vina assumes rigid target proteins and flexible ligands. According to the binding sites of fatty acids, the search area was divided into six parts for seven fatty-acid (FA) binding sites: FA1, FA2, FA3/4, FA5, FA6, and FA7. The 9 best binding modes were selected based on energy minimization [10]. The search-box settings for 1E7G, 1AO6, and 4E99 are shown in Table S2.1, S2.2, and S2.3. The docking results are constructed with two types of files: one contains free energy values corresponding to variance of the best binding modes in *.txt, and the second contains structural conformations in *.pdbqt corresponding to each binding mode. The structural conformations were visualized in Pymol software. Data cleaning to compile each energy value output into an integrated list was completed using the dataframe package in Python. The equilibrium association constant (KA) was calculated according to Equation (2) [11], where △G is the Gibb’s free energy value, and T is the temperature (in Vina, T=300 K) [12].

$$K_A = e^{-\frac{\Delta G}{RT}} \qquad Equation\ (2)$$

The precision and accuracy of the docking simulation was evaluated following the workflow outlined by Ng and Hungerbuehler (2015) [12]. The precision of the model was evaluated by repeating the docking simulations of 26 PFAS docking 1E7G at six binding

boxes for 100 times. High reproducibility was observed (Figure S2.2). Accuracy was evaluated by comparing molecular docking predictions to experimentally determine association constants as well as by redocking PFOS to HSA 4E99. 4E99 is a literature-reported HSA crystal structure complexed with PFOS [13]. First, we artificially removed PFOS and then assessed the alignment of simulated docking result for PFOS and 4E99. The atomic root mean square deviation (RMSD) between the PFOS conformation generated from docking and the experimentally obtained conformation of PFOS in 4E99 was calculated using a pairwise fitting program in PyMol (v2.3.3). A RMSD < 2 Å in this study was considered a successful redocking.

S3.2 Details on ligand preparation

We generated the 3D structures of 26 target PFAS, 18 qualified PFAS, and 18 hydrocarbon surfactnts based on their SMILES code (Table S6.2 and 6.3), using "Generating Conformers" function in DataWarrior V5.2.1 [13]. We generated the structures (one structure per stereoisomer) with the Random, low energy bias algorithm (energy minimized with MMFF94s+ forcefield), which is an optimized energy minimization method that corrected unrealistic torsion parameterization of the original MMFF94s implementation [14]. This process advanced our chance to achieve minimum energy geometry of the ligand, as this optimization process is limited by the accuracy of the starting structures (steepest descent) [15]. Then, we optimized ligand structures through molecular mechanics using MMFF94s forcefield in Avogadro V1.90.0 [16]. Finally, we used UCSF Chimera (v1.13.1)[17] 'Prep Dock' function to generate *.pdbqt, file type ready for docking.

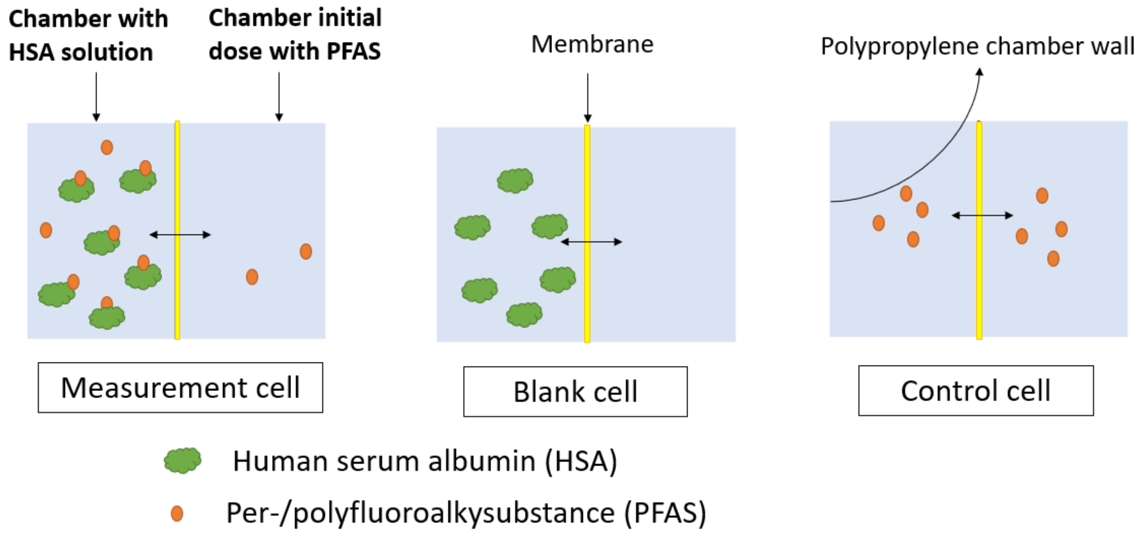

Figure S1.1 Experimental setup for equilibrium dialysis. For each trial of the experiment (n = 4), six measurement cells were prepared to determine association constants. Blank cell was used a as negative control to check contamination and membrane fouling. Control cell was used to check equilibrium and surface adsorption of equilibrium dialysis set-up. The scheme was adapted and adjusted accordingly from the published supplementary information by Allendorf et al [19]..

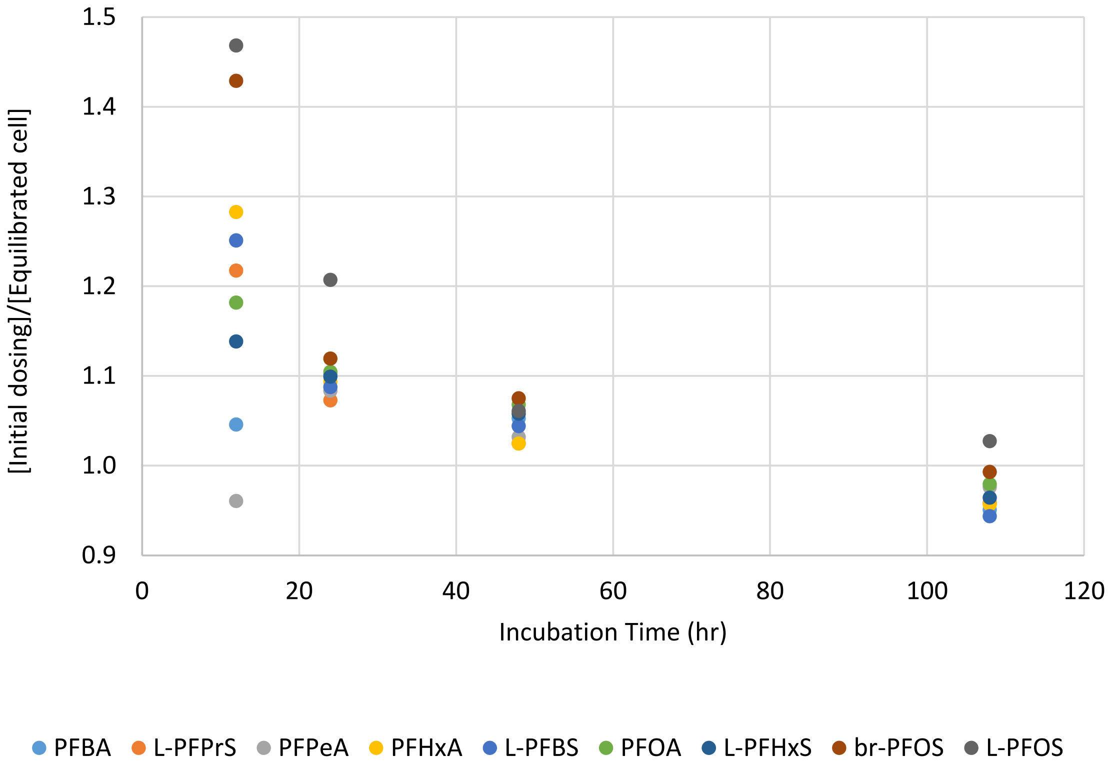

Figure S1.2 Assessment of equilibration time required for equilibrium dialysis. Equilibrium dialysis was performed with PFAS standards in phosphate buffer saline (PBS) at pH=7.4, 37 °C.

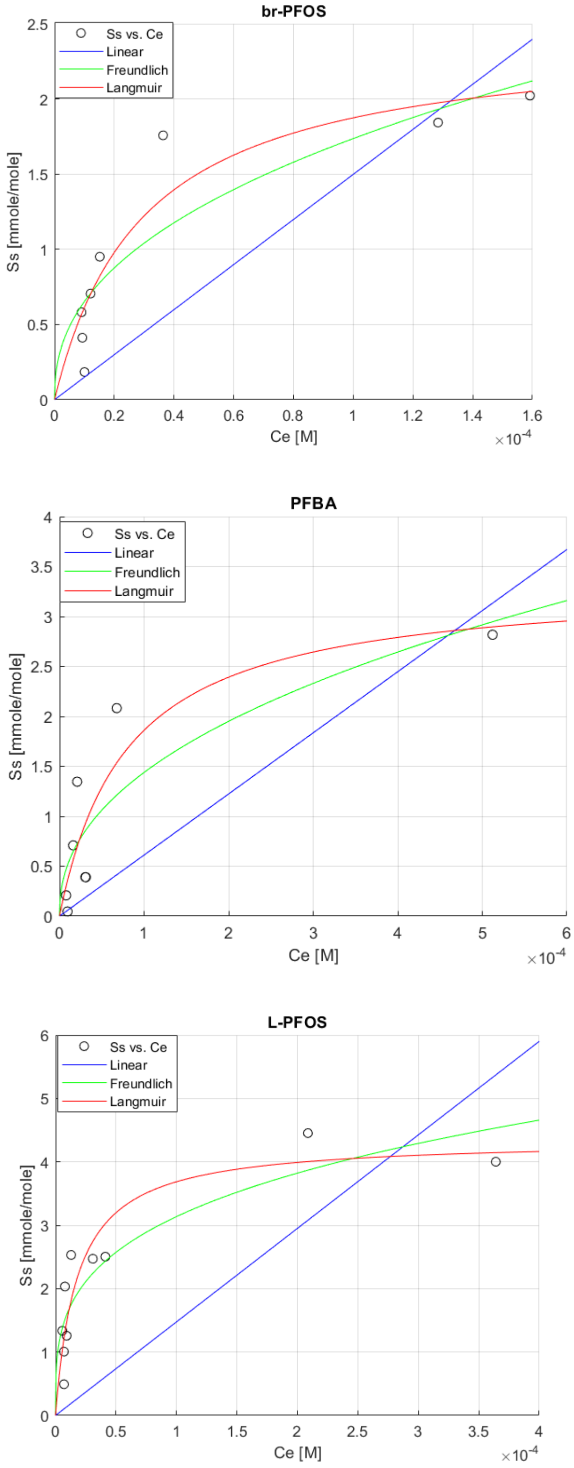

Figure S1.3 Isotherm models for PFAS adsorbed to HSA. PFBA, L-PFOS and br-PFOS in AFFF dilutions were extracted from the protein aliquot fractions and the aqueous fractions in equilibrium dialysis experiments. Fits of the bound PFAS (Ss) versus the free PFAS concentration (Ce) are shown for Linear, Freundlich, and Langmuir isotherms.

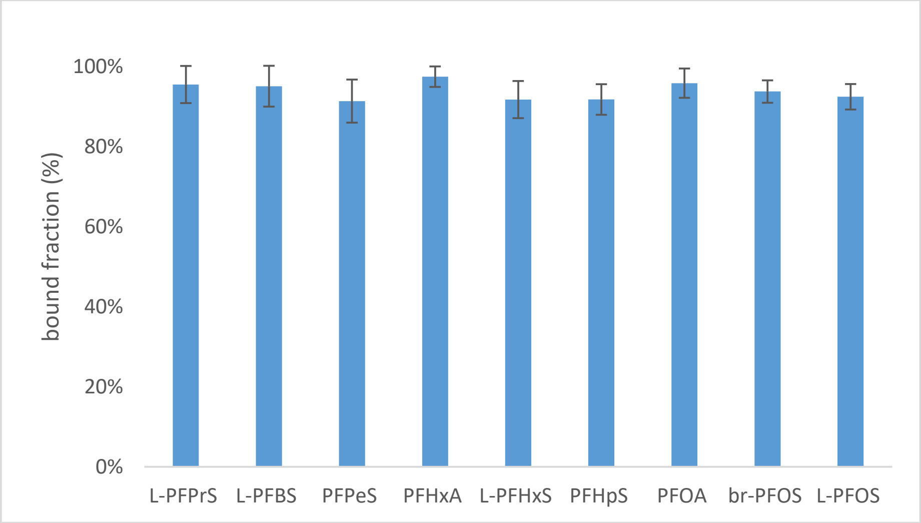

Figure S1.4 Bound fractions of PFAS compounds in AFFF (df=1E4) to HSA (600 μM). The error bar represents one standard deviation for results obtained from three batches.

a)

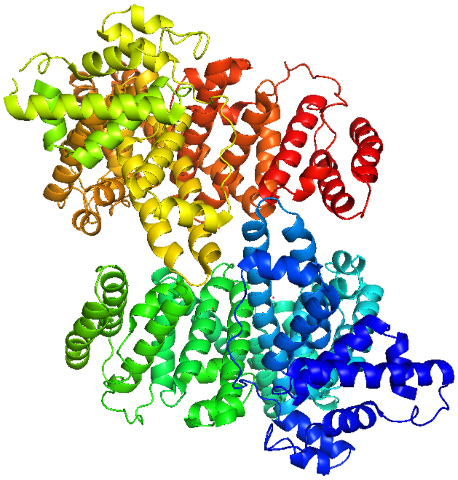

b)

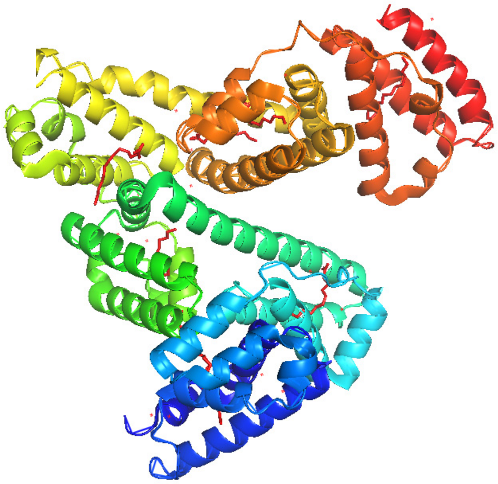

Figure S2.1 Crystal structures of Human Serum Albumin (HSA). Protein Data Bank (PDB) structures are shown for (a) 1E7G and (b) 1AO6.

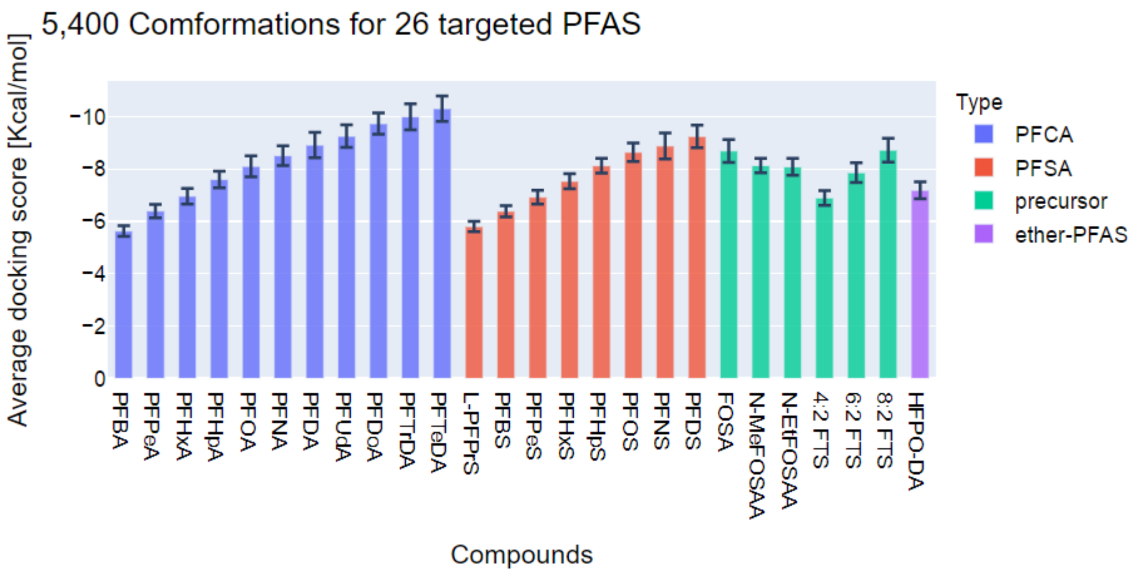

Figure S2.2 Predicted binding scores of 26-PFAS binding to HSA (1E7G).

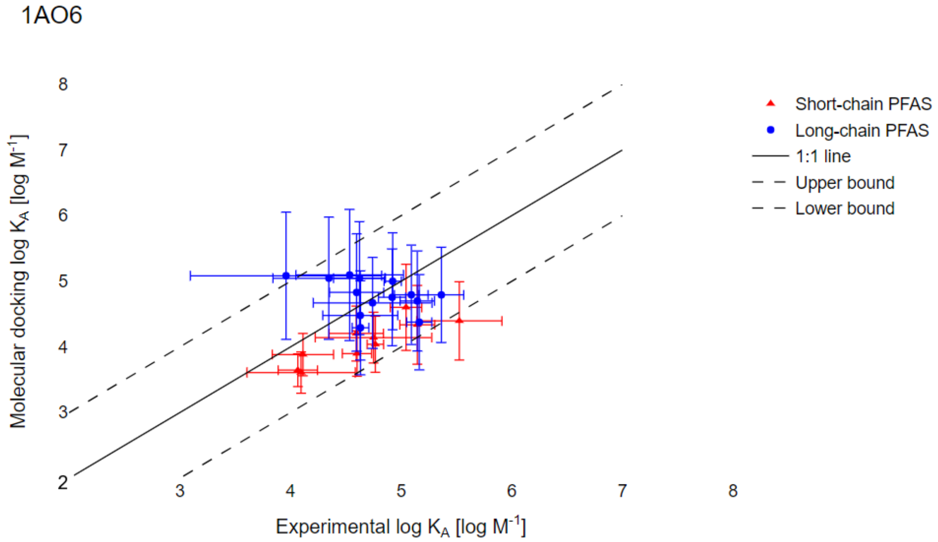

Figure S2.3. Molecular docking predicted association constants (K_A) of 26 PFAS to HSA. The comparison of experimental log K_A with results from molecular simulations was performed with HSA crystal structure 1AO6. Solid black lines represent the 1:1 line; dotted lines represent one log unit higher or lower. Error bars reflect one geometric standard deviation (GSD).

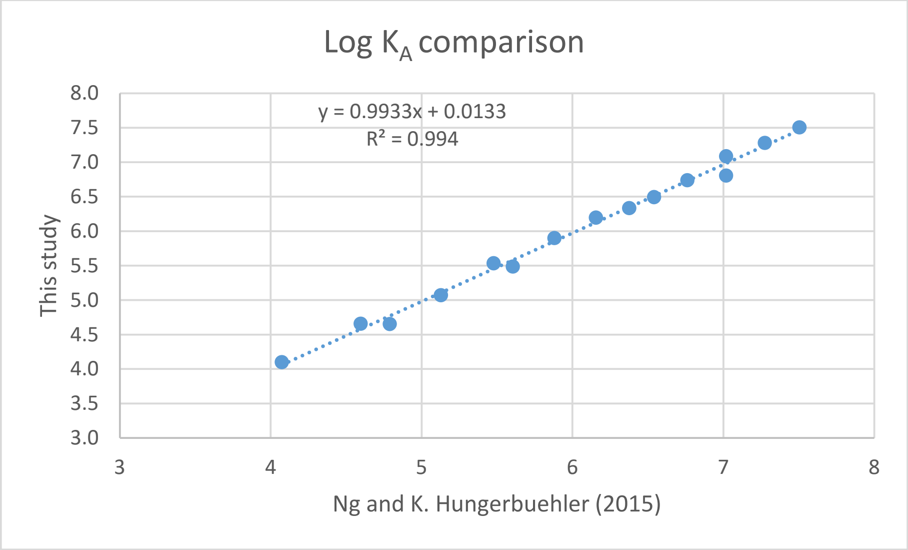

Figure S2.4 Uncorrected K_A values for 1E7G from this study correlate to the results reported by Ng and Hungerbuehler (2015). The literature reported values were extracted from the reference Supporting Information Figure S4(A).

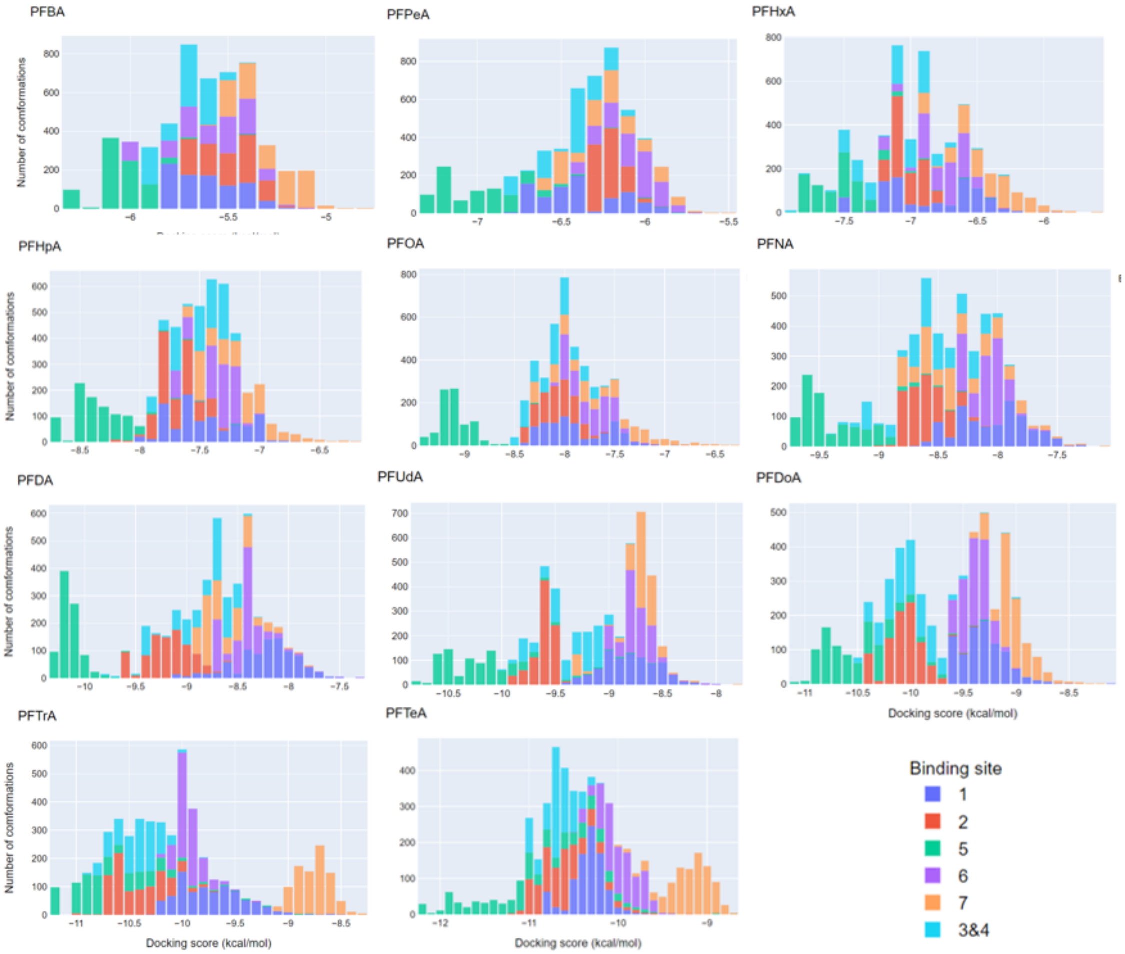

Figure S3.1 Histograms of molecular docking predicted target PFCA-1E7G binding scores.

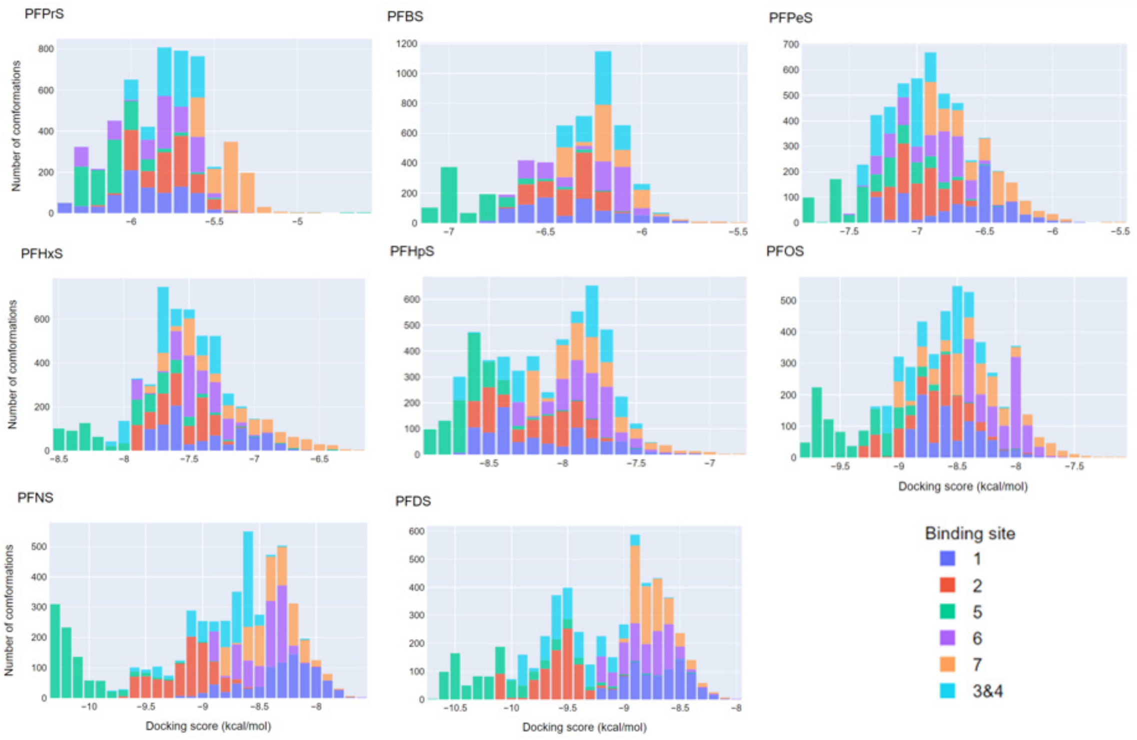

Figure S3.2 Histograms of molecular docking predicted target PFSA-1E7G binding scores.

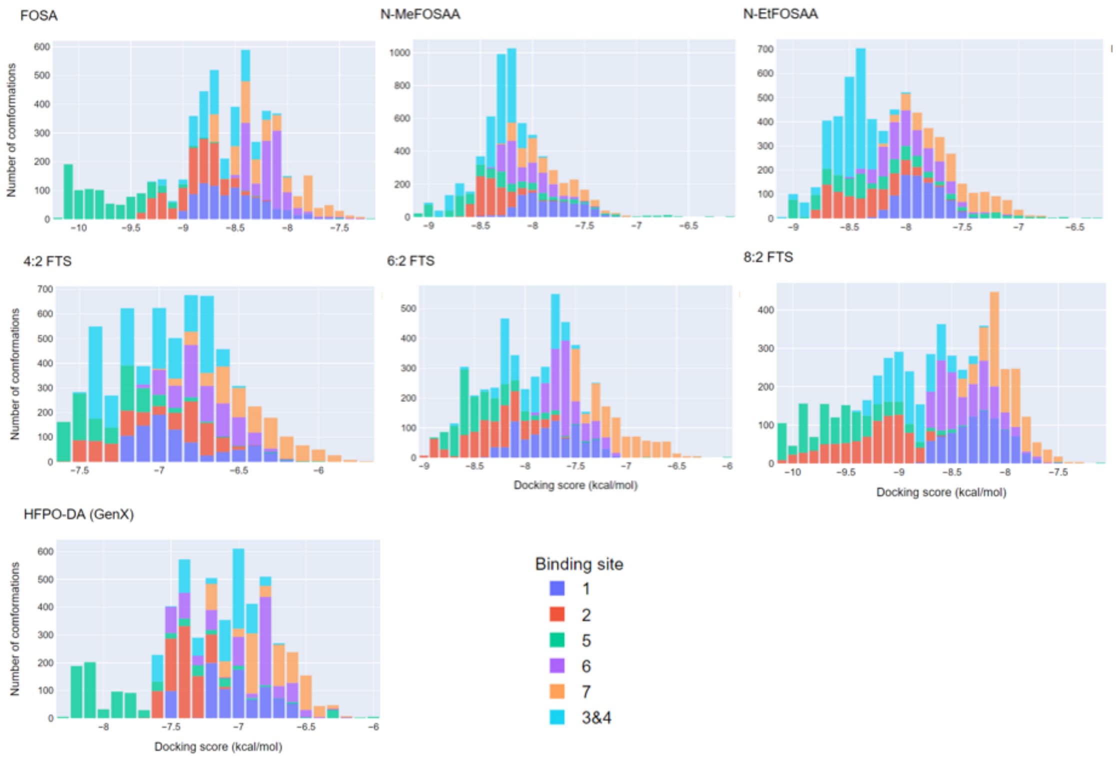

Figure S3.3 Histograms of molecular docking predicted target PFAS (precursors)-1E7G binding scores.

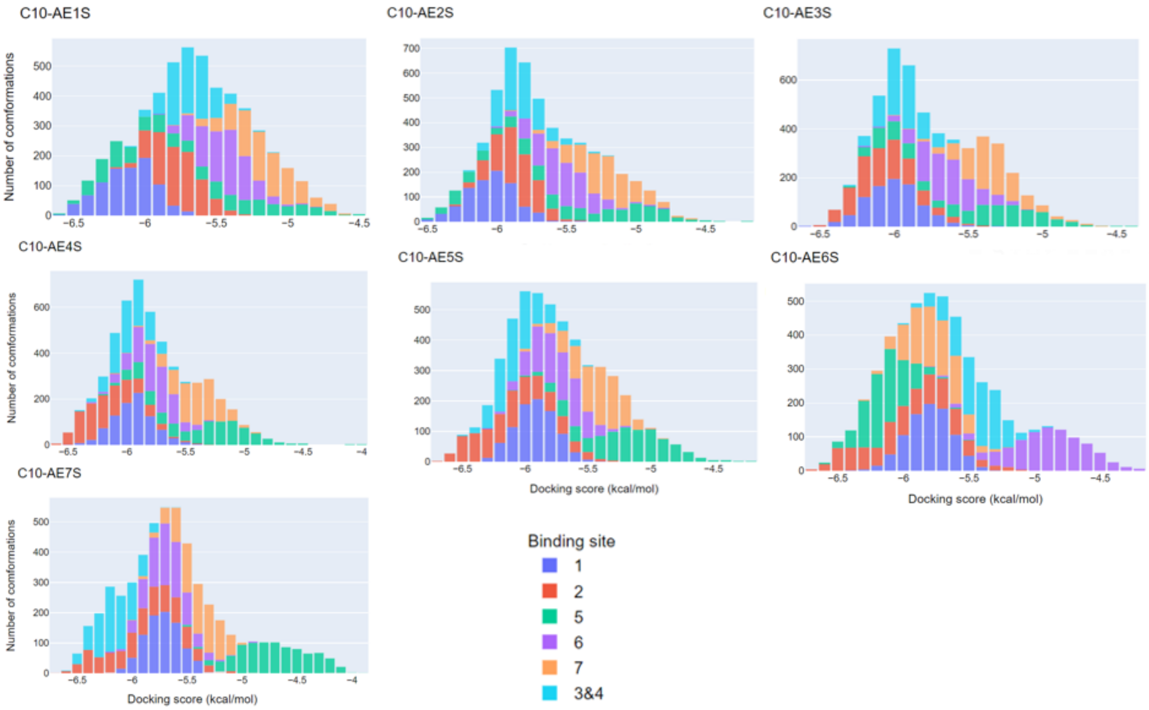

Figure S3.4 Histograms of molecular docking predicted scores for C10-AEnS binding 1E7G.

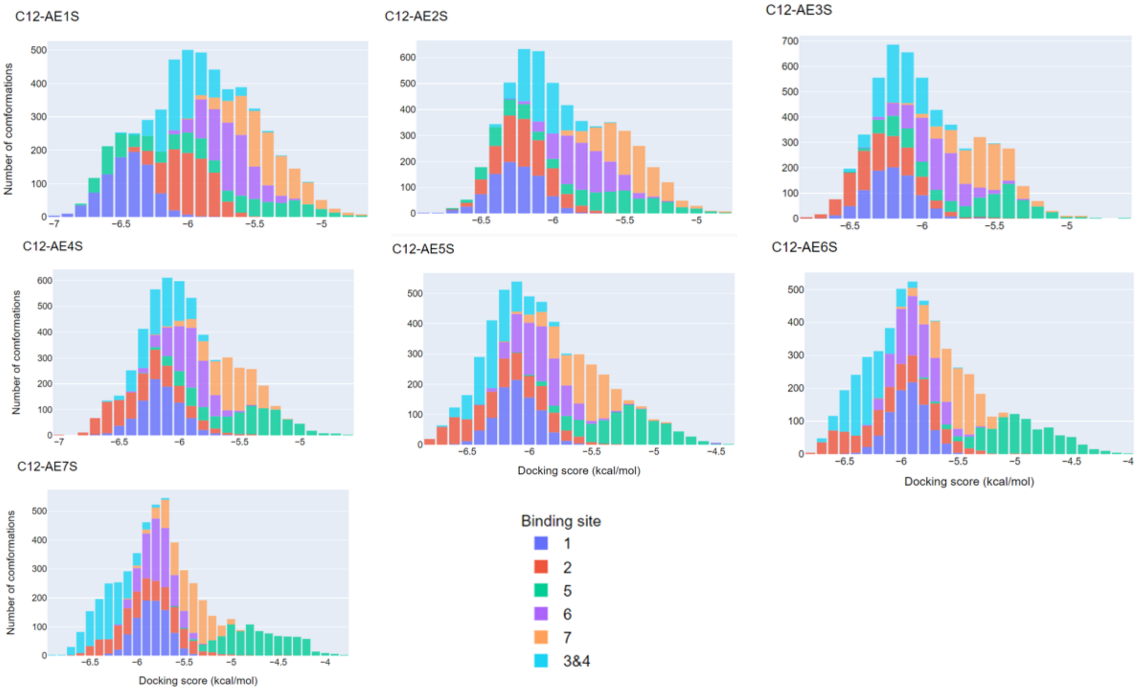

Figure S3.5 Histograms of molecular docking predicted scores for C12-AEnS binding 1E7G.

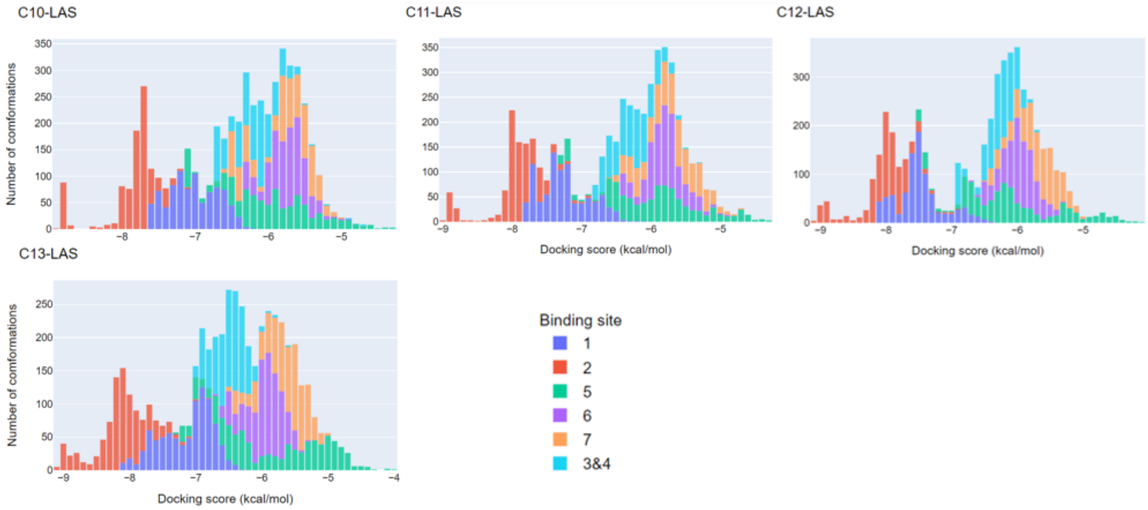

Figure S3.6 Histograms of molecular docking predicted scores for Cn-LAS binding 1E7G.

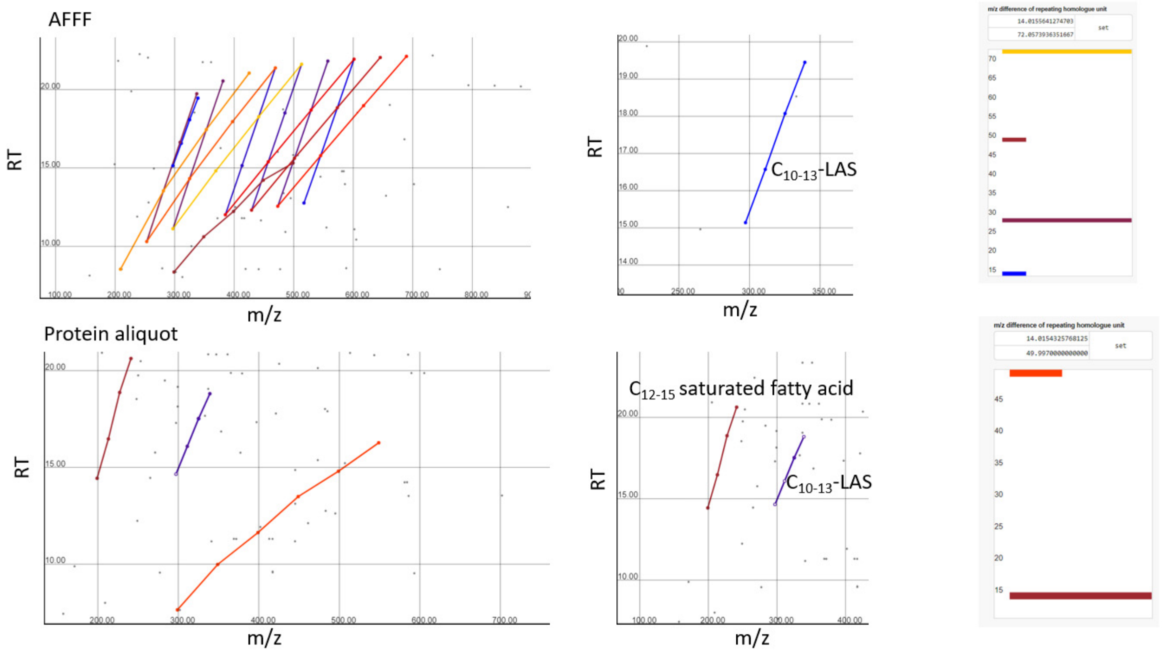

Figure S4.0 Plot of the retention time (RT) against the mass-to-charge ratio (m/z) in negative ionization mode prepared using the homologous series detection tool EnviHomolog (http://www.envihomolog.eawag.ch) across all AFFF dilutions (top) and protein aliquots (bottom). Each point represents a molecular feature; gray points were not identified as part of a homologous series while each colored line connects several points that are part of a homologous series. Colors indicate the mass of the repeating polymeric unit as shown on the inset on the right. Repeating mass-increments of 14.0156 (-CH_2-), 28.0313 (-C_2H_4-), and 44.0262 (-C_2H_4O-) were observed in dark red and bright blue colors.

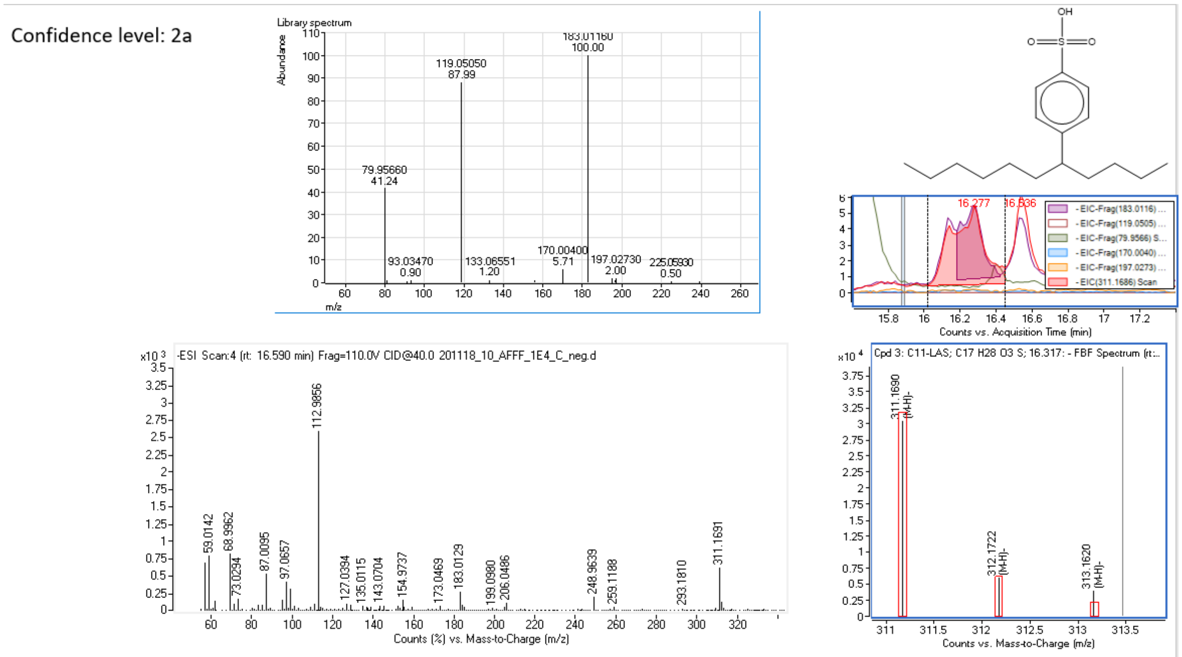

Figure S4.1 Qualification of Cn-LAS to Confidence Level 2a by matching the MONA library MS/MS spectra in ESI-. The lower left figure shows an example of the mass spectra for 40eV collision energy acquisition at the retention time (rt) shown. The top left figure shows the Massbank of North America (MONA) spectra for the suspected compound (shown at top right, C11-LAS acquired at 90eV). The middle right figure shows the chromatogram of fragment ions pulled from the MONA spectra. The lower right corner represents the isotopic pattern for the molecular ion (in black) in comparison to the theoretical isotopic pattern for the molecular formula (in red).

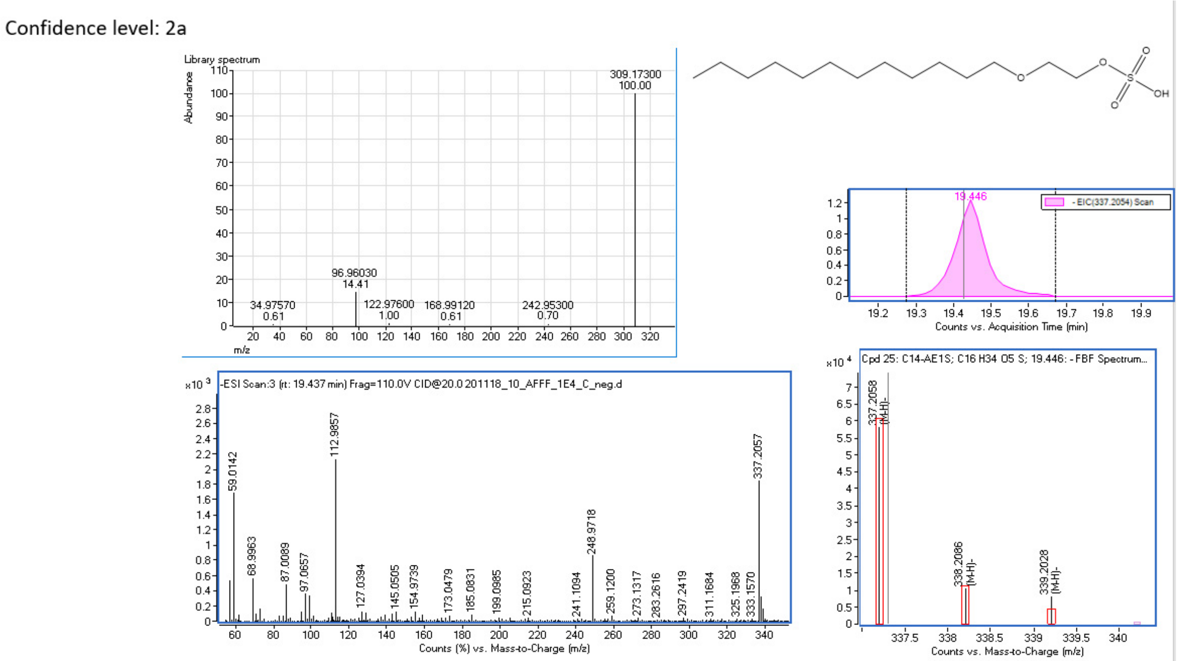

Figure S4.2 Qualification of Cn-AES to Confidence Level 2a by matching the MONA library MS/MS spectra in ESI-. The lower left figure shows an example of the mass spectra for 20eV collision energy acquisition at the retention time (rt) shown. The top left figure shows the Massbank of North America (MONA) spectra for the suspected compound (shown at top right, C12-AES acquired at 60eV). The middle right figure shows the chromatogram of fragment ions pulled from the MONA spectra. The lower right corner represents the isotopic pattern for the molecular ion (in black) in comparison to the theoretical isotopic pattern for the molecular formula (in red).

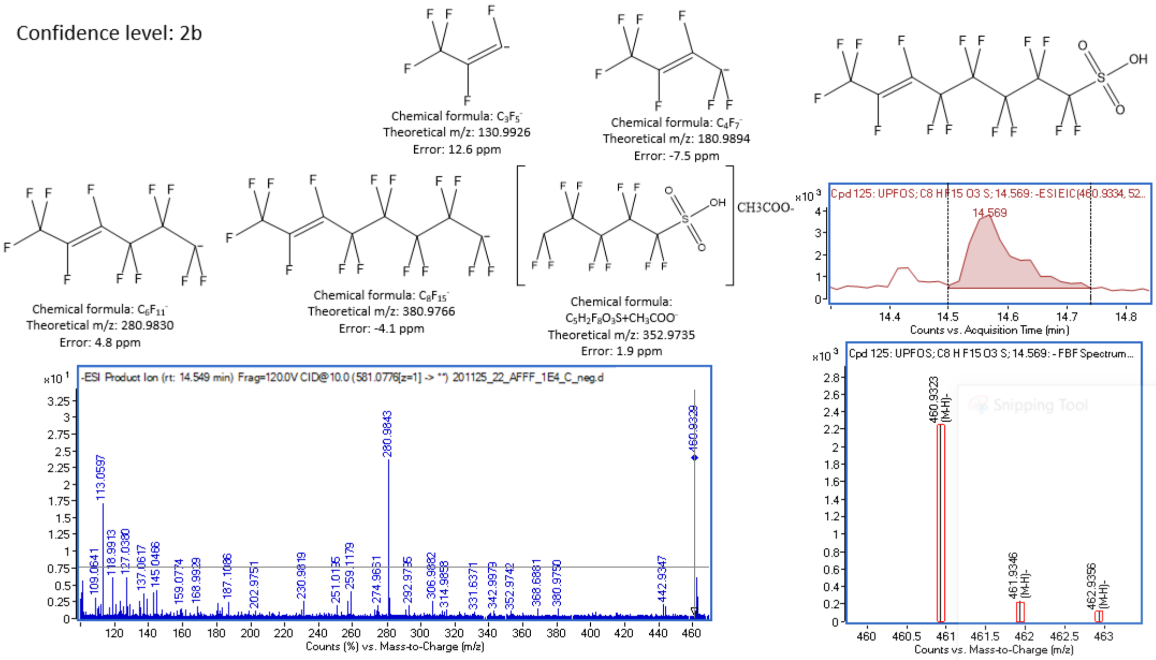

Figure S4.3 Qualification of UPFOS to Confidence Level 2b in ESI-. Structures for the qualified compound and all fragments ions detected are shown. The middle right inset shows the extracted chromatogram for the molecular ion. The lower right inset figure shows the isotopic pattern (in black) in comparison to the theoretical isotopic pattern for the molecular formula (in red). The lower left figure shows an example of the mass spectra for the targeted MS/MS of the AFFF sample (df = 1E4) at 10eV collision energy acquisition at the retention time (rt) shown.

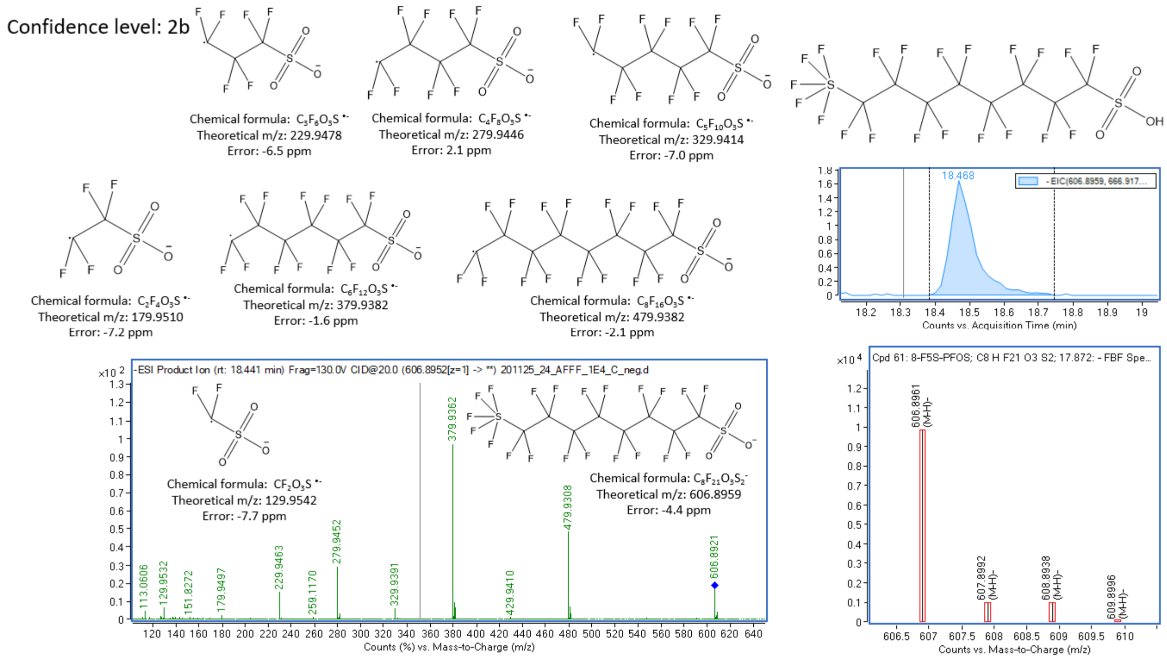

Figure S4.4 Qualification of 8-F5S-PFOS to Confidence Level 2b in ESI-. Structures for the qualified compound and all fragments ions detected are shown. The middle right inset shows the extracted chromatogram for the molecular ion. The lower right inset figure shows the isotopic pattern (in black) in comparison to the theoretical isotopic pattern for the molecular formula (in red). The lower left figure shows an example of the mass spectra for the targeted MS/MS of the AFFF sample (df = 1E4) at 20eV collision energy acquisition at the retention time (rt) shown.

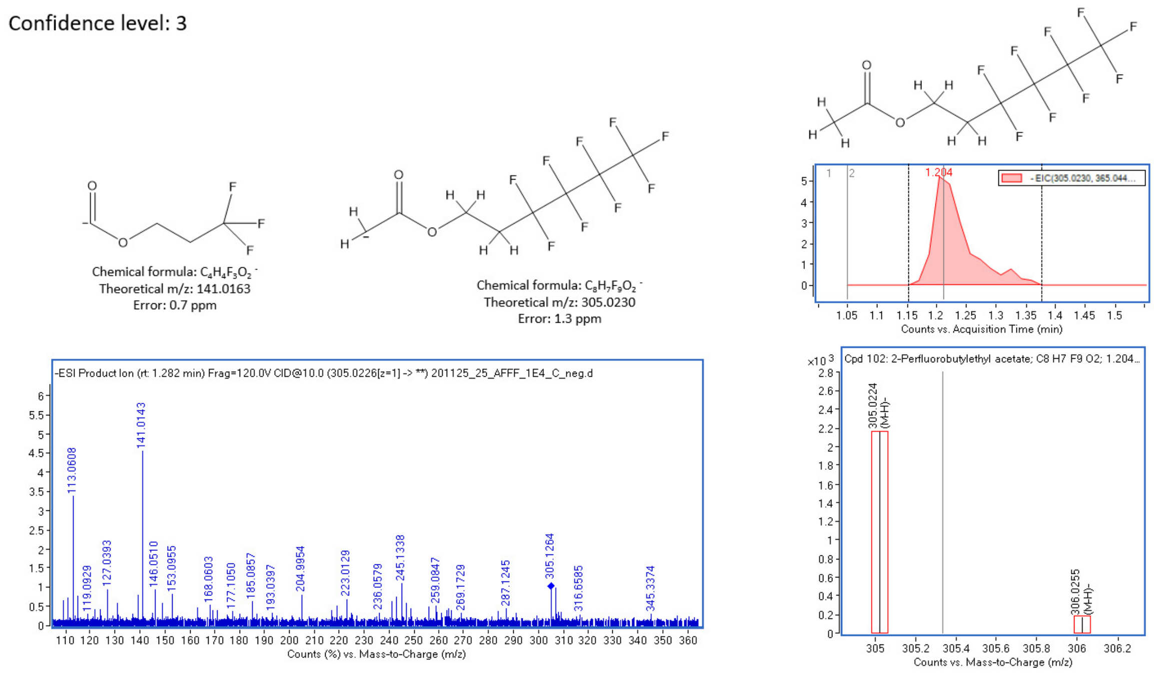

Figure S4.5 Qualification of N-FBEAc to Confidence Level 3 in ESI-. Structures for the qualified compound and all fragments ions detected are shown. The middle right inset shows the extracted chromatogram for the molecular ion. The lower right inset figure shows the isotopic pattern (in black) in comparison to the theoretical isotopic pattern for the molecular formula (in red). The lower left figure shows an example of the mass spectra for the targeted MS/MS of the AFFF sample (df = 1E4) at 10eV collision energy acquisition at the retention time (rt) shown.

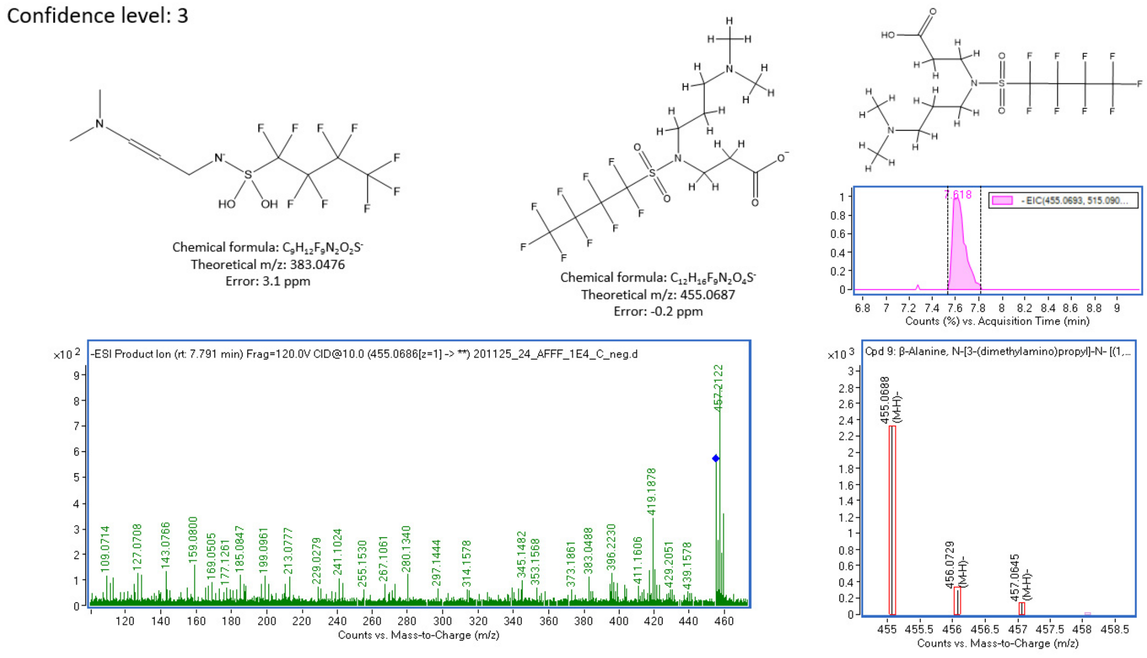

Figure S4.6 Qualification of N-diMAmP-FBSAP to Confidence Level 3 in ESI-. Structures for the qualified compound and all fragments ions detected are shown. The middle right inset shows the extracted chromatogram for the molecular ion. The lower right inset figure shows the isotopic pattern (in black) in comparison to the theoretical isotopic pattern for the molecular formula (in red). The lower left figure shows an example of the mass spectra for the targeted MS/MS of the AFFF sample (df = 1E4) at 10eV collision energy acquisition at the retention time (rt) shown.

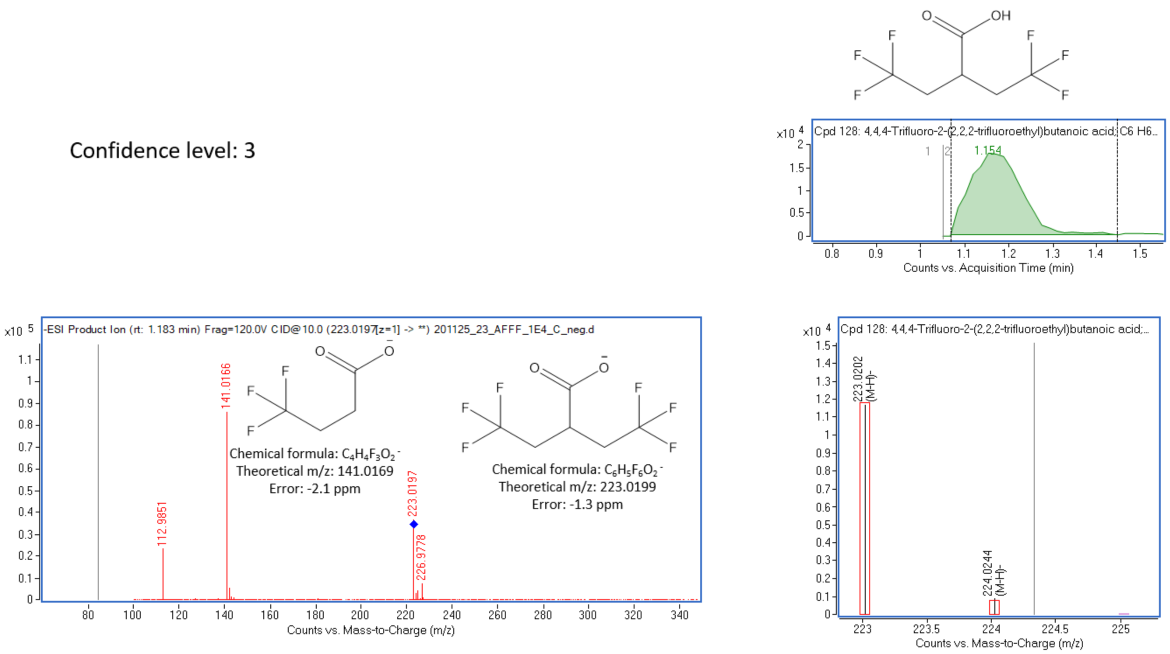

Figure S4.7 Qualification of diTF-IsoBA to Confidence Level 3 in ESI-. Structures for the qualified compound and all fragments ions detected are shown. The middle right inset shows the extracted chromatogram for the molecular ion. The lower right inset figure shows the isotopic pattern (in black) in comparison to the theoretical isotopic pattern for the molecular formula (in red). The lower left figure shows an example of the mass spectra for the targeted MS/MS of the AFFF sample (df = 1E4) at 10eV collision energy acquisition at the retention time (rt) shown.

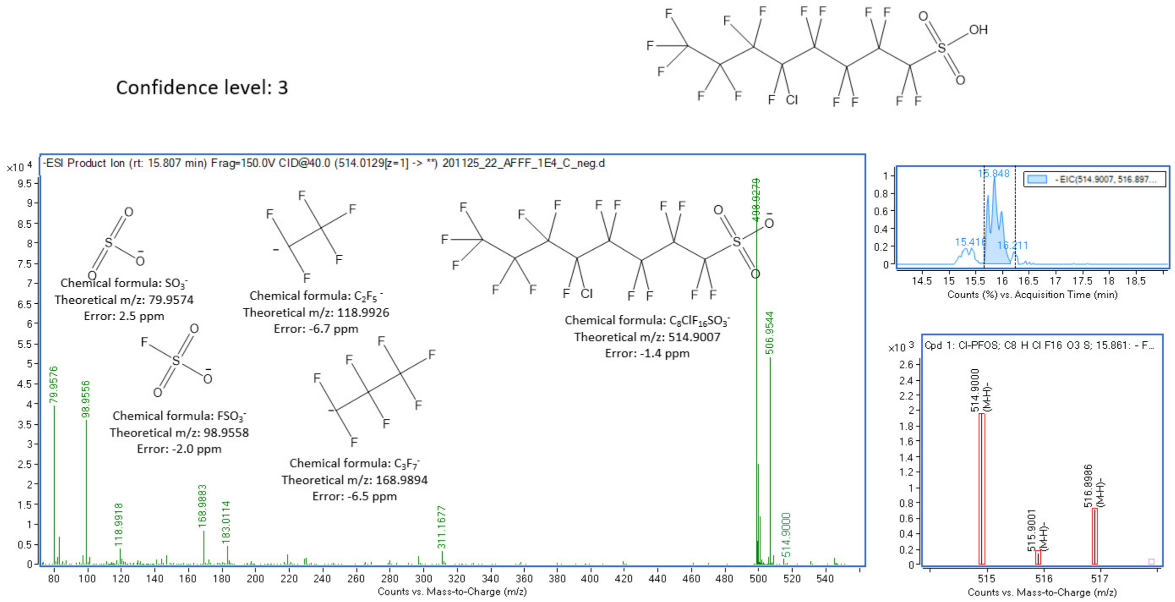

Figure S4.8 Qualification of Cl-PFOS to Confidence Level 3 in ESI-. Structures for the qualified compound and all fragments ions detected are shown. The middle right inset shows the extracted chromatogram for the molecular ion. The lower right inset figure shows the isotopic pattern (in black) in comparison to the theoretical isotopic pattern for the molecular formula (in red). The lower left figure shows an example of the mass spectra for the targeted MS/MS of the AFFF sample (df = 1E4) at 40eV collision energy acquisition at the retention time (rt) shown.

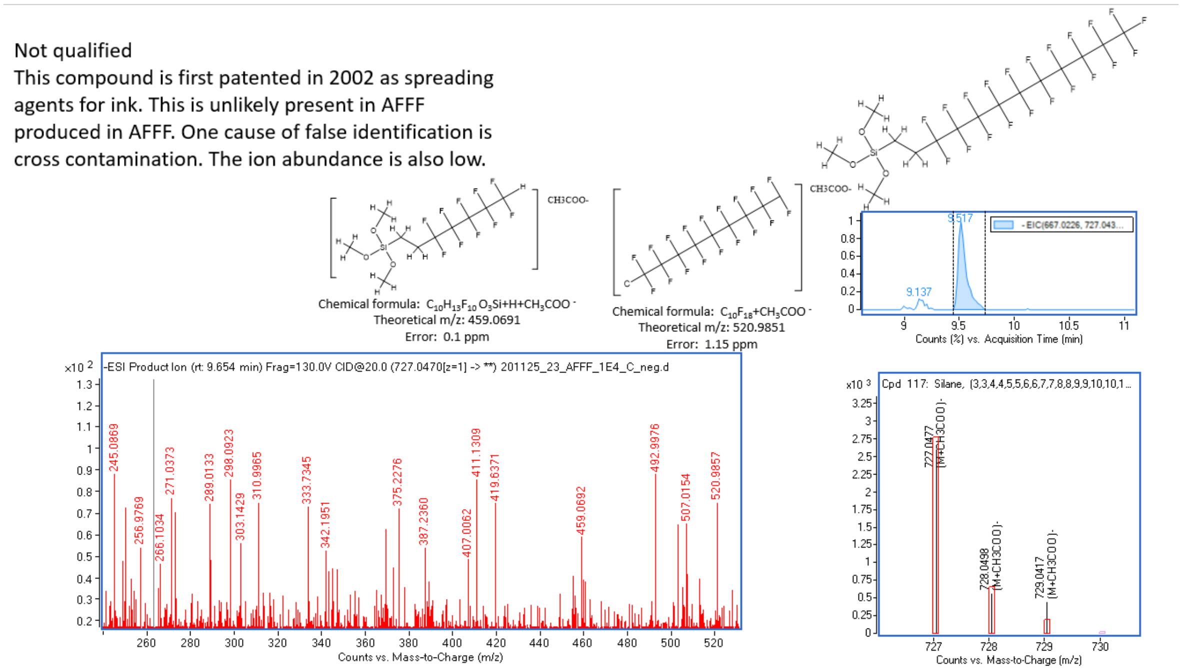

Figure S4.9 Annotated MS/MS spectrum of 1H,1H,2H,2H-Perfluorododecyltrimethoxysilane. Structures for the qualified compound and all fragments ions detected are shown. The middle right inset shows the extracted chromatogram for the molecular ion. The lower right inset figure shows the isotopic pattern (in black) in comparison to the theoretical isotopic pattern for the molecular formula (in red). The lower left figure shows an example of the mass spectra for the targeted MS/MS of the AFFF sample (df = 1E4) at 20eV collision energy acquisition at the retention time (rt) shown.

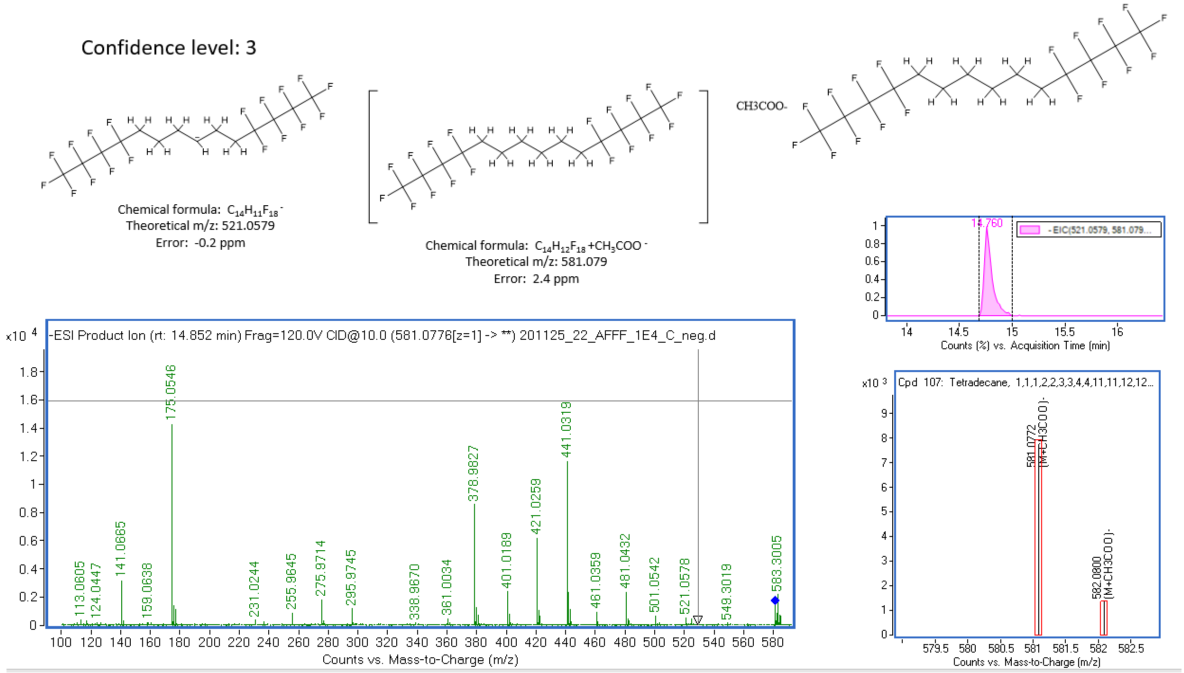

Figure S4.10 Qualification of Hx-diFB to Confidence Level 3 in ESI-. Structures for the qualified compound and all fragments ions detected are shown. The middle right inset shows the extracted chromatogram for the molecular ion. The lower right inset figure shows the isotopic pattern (in black) in comparison to the theoretical isotopic pattern for the molecular formula (in red). The lower left figure shows an example of the mass spectra for the targeted MS/MS of the AFFF sample (df = 1E4) at 10eV collision energy acquisition at the retention time (rt) shown.

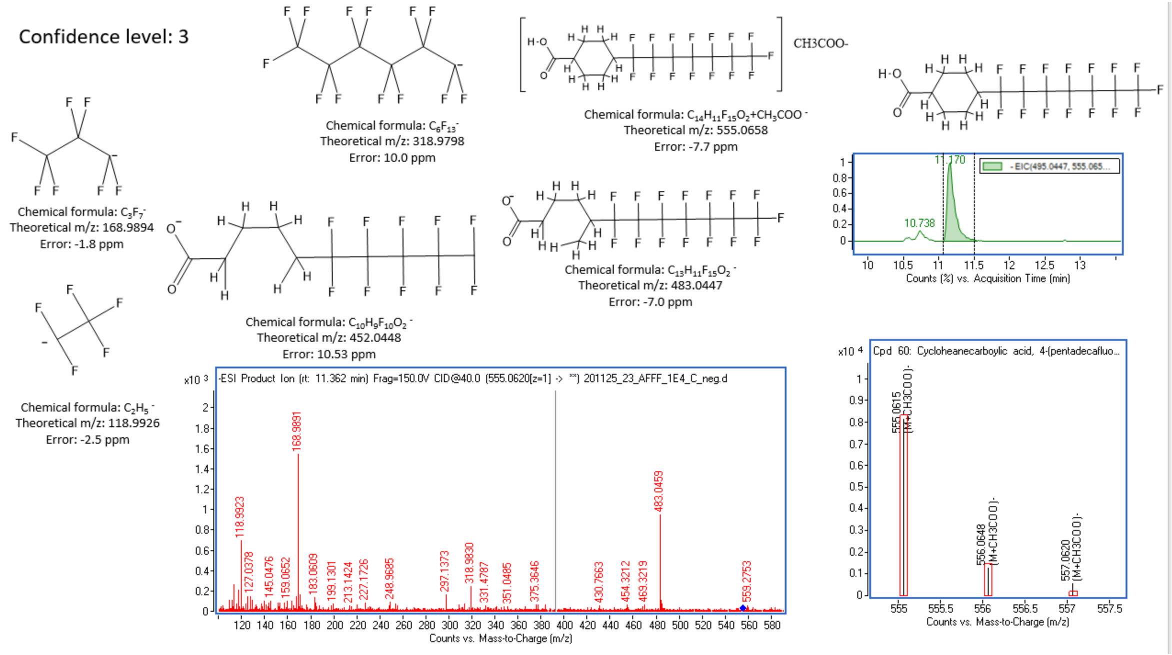

Figure S4.11 Qualification of 4-FHp-CycHxA to Confidence Level 3 in ESI-. Structures for the qualified compound and all fragments ions detected are shown. The middle right inset shows the extracted chromatogram for the molecular ion. The lower right inset figure shows the isotopic pattern (in black) in comparison to the theoretical isotopic pattern for the molecular formula (in red). The lower left figure shows an example of the mass spectra for the targeted MS/MS of the AFFF sample (df = 1E4) at 40eV collision energy acquisition at the retention time (rt) shown.

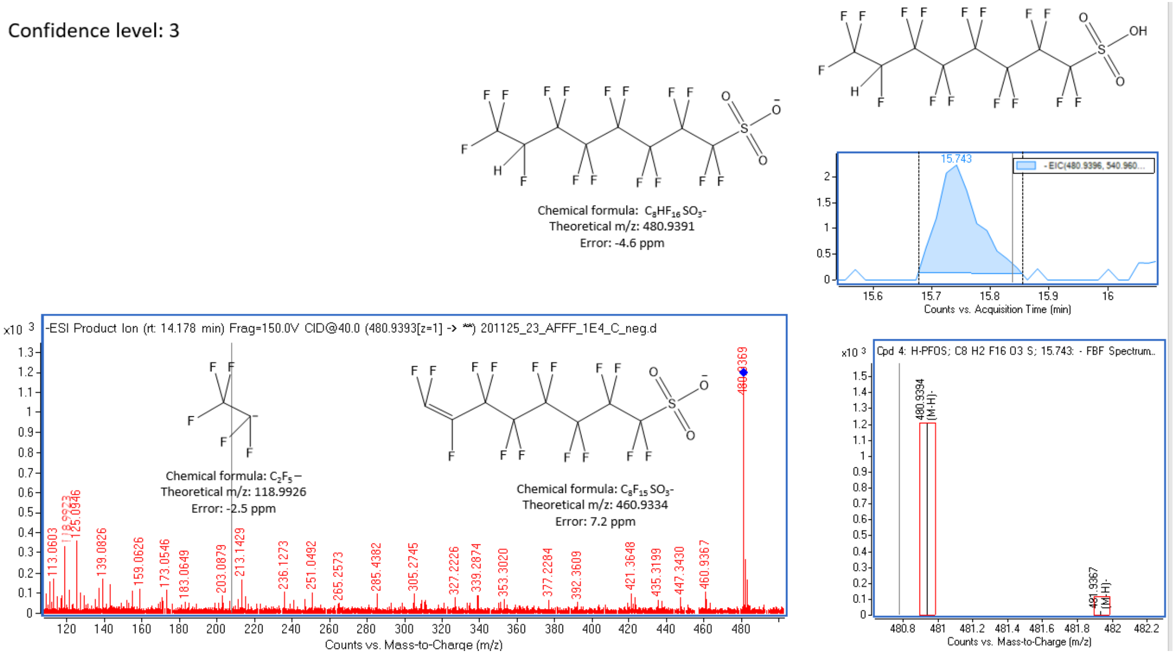

Figure S4.12 Qualification of H-PFDS to Confidence Level 3 in ESI-. Structures for the qualified compound and all fragments ions detected are shown. The middle right inset shows the extracted chromatogram for the molecular ion. The lower right inset figure shows the isotopic pattern (in black) in comparison to the theoretical isotopic pattern for the molecular formula (in red). The lower left figure shows an example of the mass spectra for the targeted MS/MS of the AFFF sample (df = 1E4) at 40eV collision energy acquisition at the retention time (rt) shown.

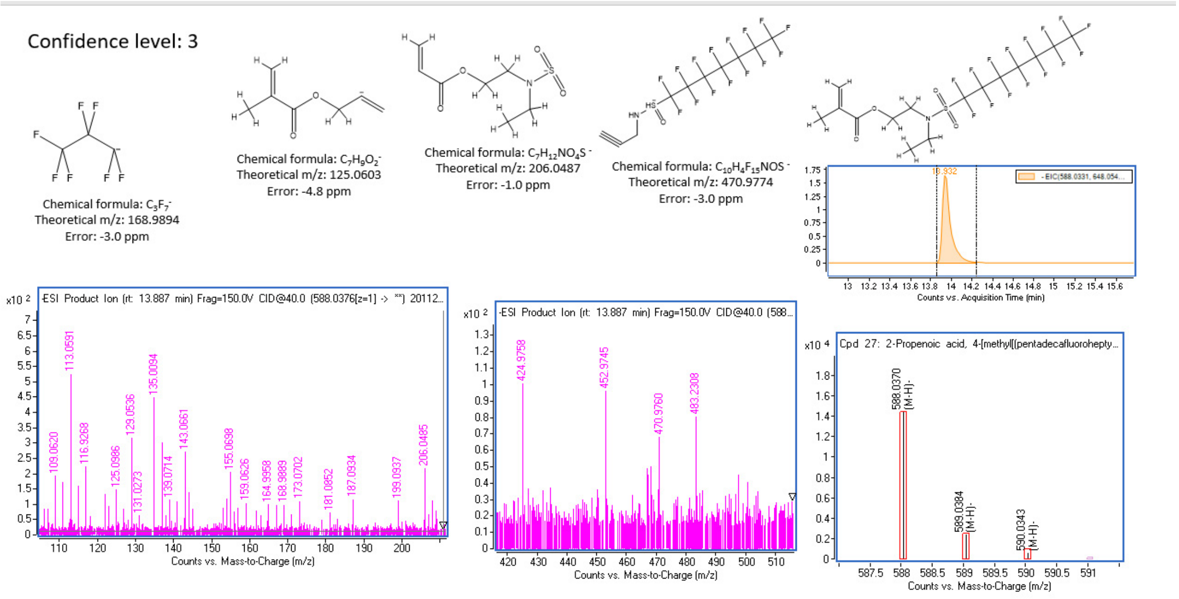

Figure S4.13 Qualification of N-EFHpSA-EMAC to Confidence Level 3 in ESI-. Structures for the qualified compound and all fragments ions detected are shown. The middle right inset shows the extracted chromatogram for the molecular ion. The lower right inset figure shows the isotopic pattern (in black) in comparison to the theoretical isotopic pattern for the molecular formula (in red). The lower left figure shows an example of the mass spectra for the targeted MS/MS of the AFFF sample (df = 1E4) at 40eV collision energy acquisition at the retention time (rt) shown.

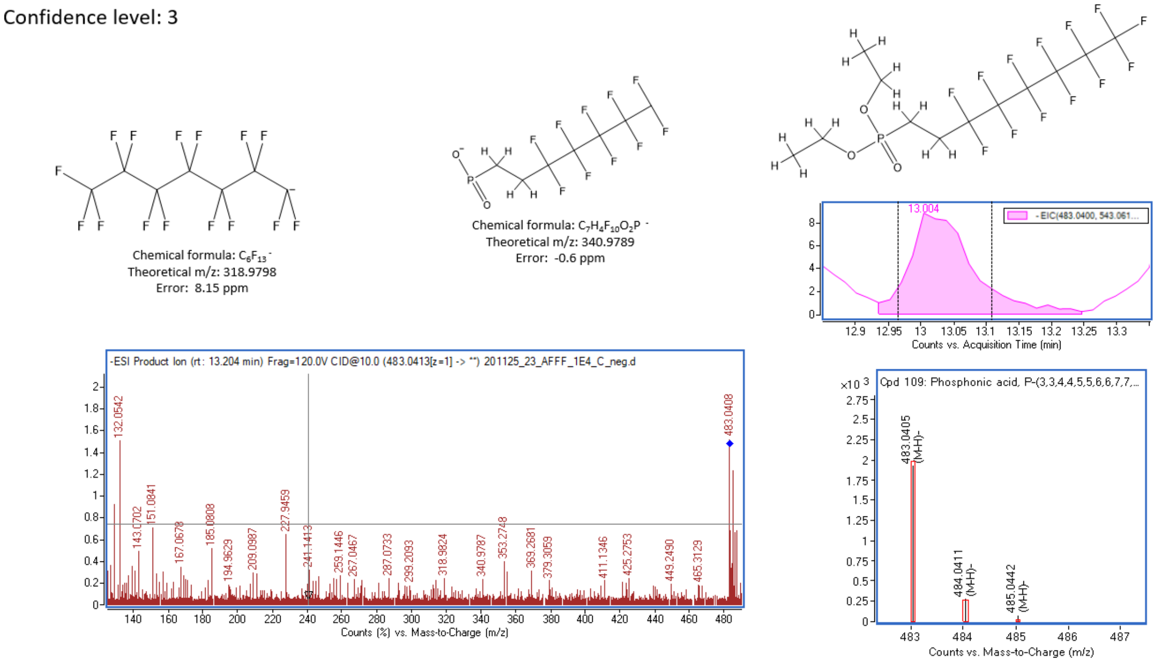

Figure S4.14 Qualification of 8:2 monoPAP-diEes to Confidence Level 3 in ESI-. Structures for the qualified compound and all fragments ions detected are shown. The middle right inset shows the extracted chromatogram for the molecular ion. The lower right inset figure shows the isotopic pattern (in black) in comparison to the theoretical isotopic pattern for the molecular formula (in red). The lower left figure shows an example of the mass spectra for the targeted MS/MS of the AFFF sample (df = 1E4) at 10eV collision energy acquisition at the retention time (rt) shown.

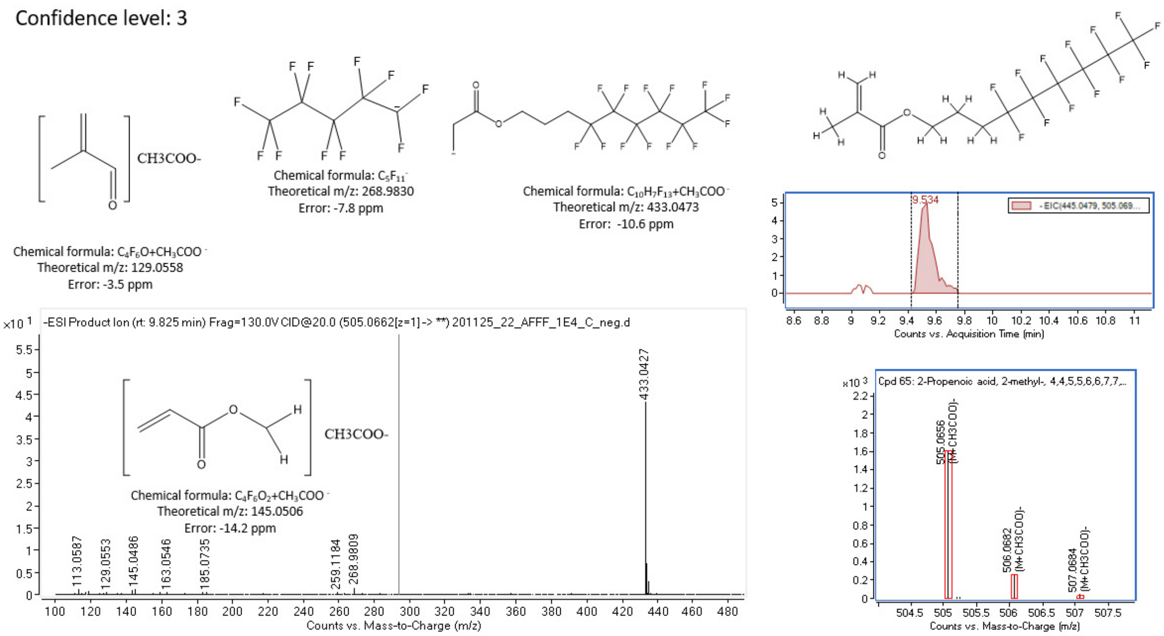

Figure S4.15 Qualification of N-FHxP-MAC to Confidence Level 3 in ESI⁻. Structures for the qualified compound and all fragments ions detected are shown. The middle right inset shows the extracted chromatogram for the molecular ion. The lower right inset figure shows the isotopic pattern (in black) in comparison to the theoretical isotopic pattern for the molecular formula (in red). The lower left figure shows an example of the mass spectra for the targeted MS/MS of the AFFF sample (df = 1E4) at 20eV collision energy acquisition at the retention time (rt) shown.

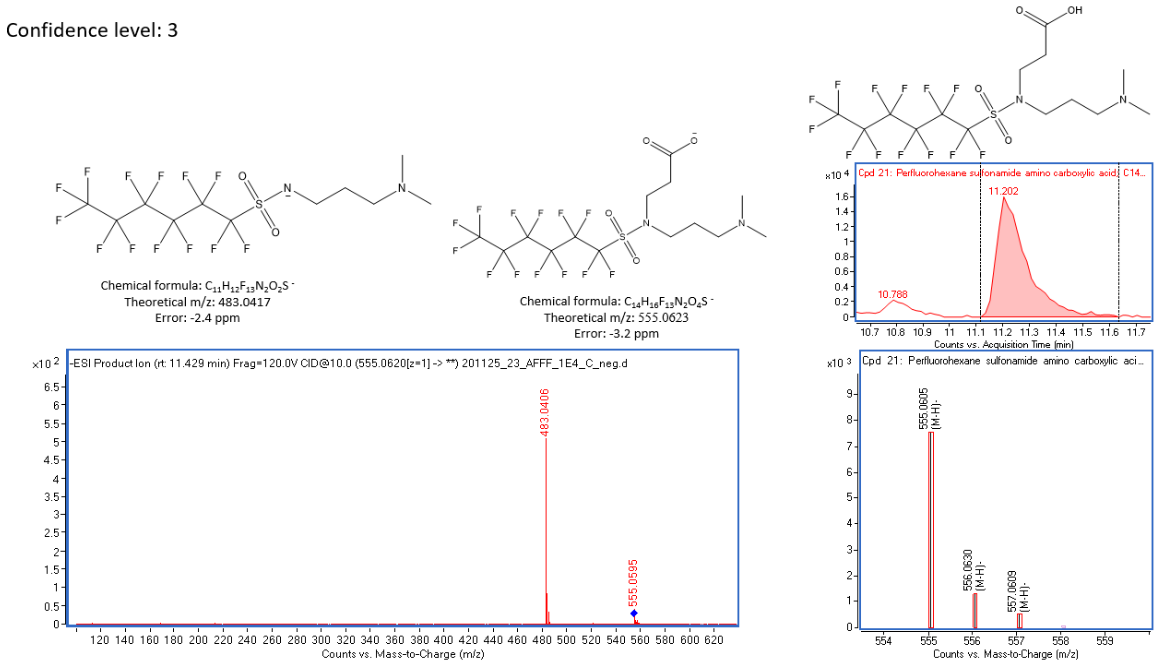

Figure S4.16 Qualification of N-TMAmP-FHxSAE to Confidence Level 3 in ESI⁻. Structures for the qualified compound and all fragments ions detected are shown. The middle right inset shows the extracted chromatogram for the molecular ion. The lower right inset figure shows the isotopic pattern (in black) in comparison to the theoretical isotopic pattern for the molecular formula (in red). The lower left figure shows an example of the mass spectra for the targeted MS/MS of the AFFF sample (df = 1E4) at 10eV collision energy acquisition at the retention time (rt) shown.

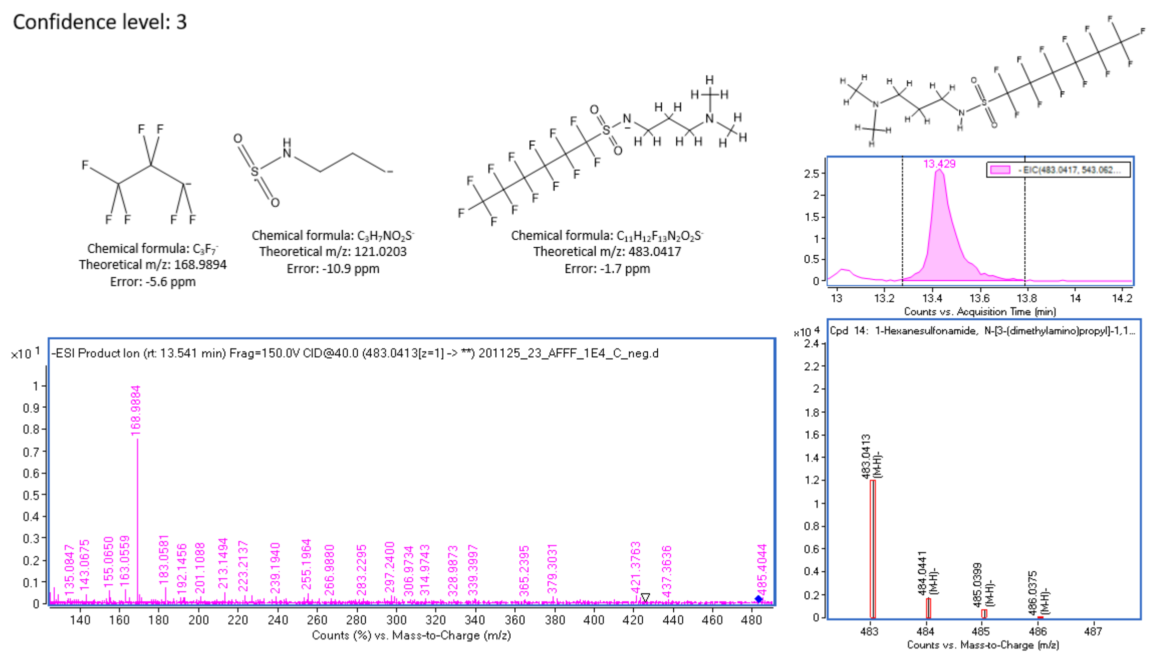

Figure S4.17 Qualification of N-diMAmP-FHxSA to Confidence Level 3 in ESI-. Structures for the qualified compound and all fragments ions detected are shown. The middle right inset shows the extracted chromatogram for the molecular ion. The lower right inset figure shows the isotopic pattern (in black) in comparison to the theoretical isotopic pattern for the molecular formula (in red). The lower left figure shows an example of the mass spectra for the targeted MS/MS of the AFFF sample (df = 1E4) at 40eV collision energy acquisition at the retention time (rt) shown.

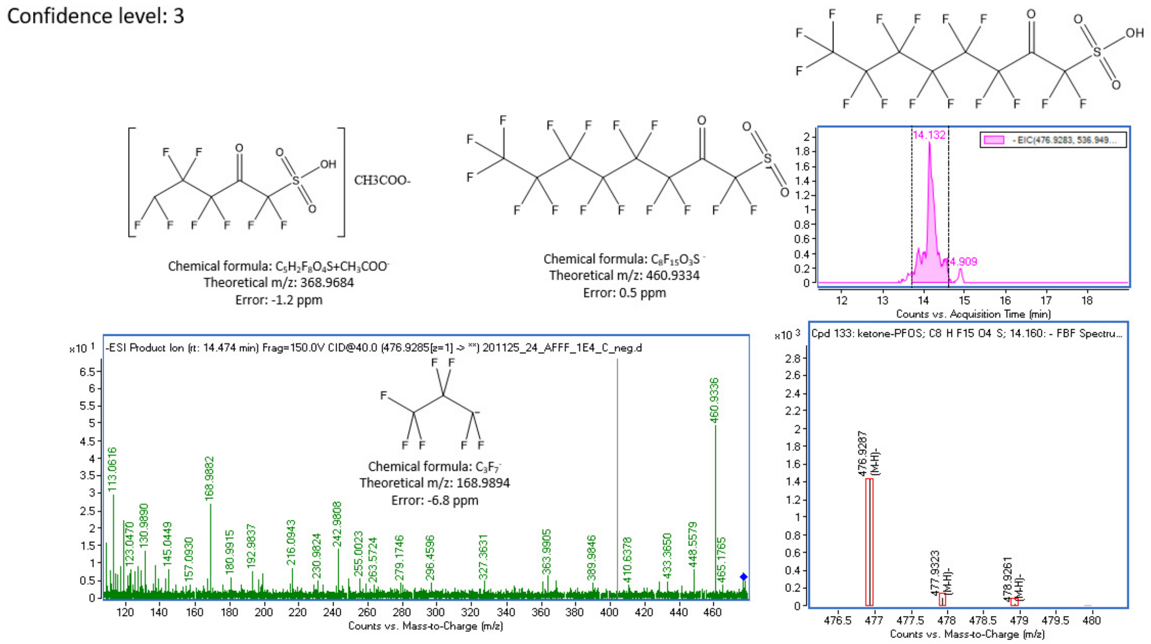

Figure S4.18 Qualification of K-PFOS to Confidence Level 3 in ESI-. Structures for the qualified compound and all fragments ions detected are shown. The middle right inset shows the extracted chromatogram for the molecular ion. The lower right inset figure shows the isotopic pattern (in black) in comparison to the theoretical isotopic pattern for the molecular formula (in red). The lower left figure shows an example of the mass spectra for the targeted MS/MS of the AFFF sample (df = 1E4) at 40eV collision energy acquisition at the retention time (rt) shown.

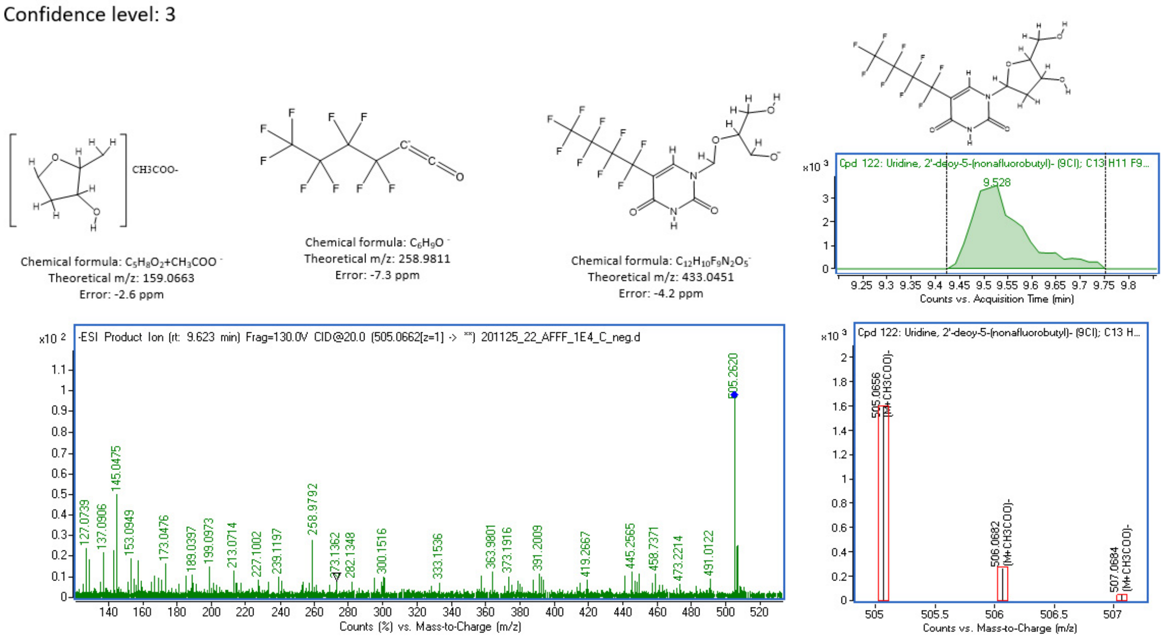

Figure S4.19 Qualification of Uridine-FB to Confidence Level 3 in ESI-. Structures for the qualified compound and all fragments ions detected are shown. The middle right inset shows the extracted chromatogram for the molecular ion. The lower right inset figure shows the isotopic pattern (in black) in comparison to the theoretical isotopic pattern for the molecular formula (in red). The lower left figure shows an example of the mass spectra for the targeted MS/MS of the AFFF sample (df = 1E4) at 20eV collision energy acquisition at the retention time (rt) shown.

Table S1.1 Mass (ng) of PFAS present in each fraction of equilibrium dialysis extracts.

**PFAS dosage**	**Covalent**			**Non-covalent**			Aqueous portion		
	**PFBS**	**PFOA**	**PFOS**	**PFBS**	**PFOA**	PFOS	PFBS	PFOA	PFOS
26 PFAS [200 ng]	3.91	20.25	3.70	110.95	51.50	206.63	11.0	3.1	6.8
26 PFAS [80 ng]	6.12	16.10	3.64	52.19	50.39	61.77	6.4	1.8	6.3
26 PFAS [40 ng]	8.29	3.57	3.26	21.00	28.26	31.25	6.6	2.0	7.7
26 PFAS [20 ng]	13.22	7.43	3.20	13.40	18.74	8.77	<LOQ	1.4	15.3
26 PFAS [10 ng]	8.22	4.36	3.27	4.81	9.73	6.11	7.2	2.6	7.3
26 PFAS [4 ng]	2.61	0.95	3.16	4.89	11.19	2.57	<LOQ	<LOQ	6.5
AFFF (df=2e3)	2.43	17.34	4.50	9.69	10.20	584.25	6.2	2.3	6.4
AFFF (df=4e3)	1.18	12.95	3.87	9.98	8.96	267.56	<LOQ	<LOQ	6.5
AFFF (df=1.6e4)	1.72	15.10	3.25	5.36	8.68	121.98	<LOQ	<LOQ	7.8
AFFF (df=2e4)	4.56	20.42	3.54	9.50	4.24	95.45	<LOQ	0.5	7.2
AFFF (df=4e4)	0.88	22.25	3.27	5.85	7.88	53.11	<LOQ	<LOQ	7.1
AFFF (df=8e4)	0.90	21.84	3.35	5.41	9.87	16.10	<LOQ	<LOQ	7.1

Table S2. 1 Molecular docking searching information for 1E7G.

**Binding Sites**	**Search Center(Å)**			**Size of Search Box(Å)**		
	**x**	**y**	**z**	**x**	**y**	**z**
FA1	32.913	15.789	32.677	18	20	30
FA2	47.386	9.578	20.034	20	24	22
FA3/4	11.193	5.903	20.079	22	28	22
FA5	0.702	5.791	42.116	20	20	22
FA6	24.958	9.578	1.116	20	22	22
FA7	33.244	10.42	15.488	20	22	20

Table S2. 2 Molecular docking searching information for 1AO6.

Binding Sites	Search Center(Å)			Size of Search Box(Å)		
	x	y	z	x	y	z
FA1	45	25	18	28	28	34
FA2	50	45	18	32	25	33
FA3/4	20	30	20	20	35	25
FA5	18	20	0	28	20	25
FA6	10	40	45	23	25	25
FA7	35	35	40	30	25	25

Table S2. 3 Molecular docking searching information for 4E99.

Binding Sites	Search Center(Å)			Size of Search Box(Å)			RMSD range for 9 poses (Å)
	x	y	z	x	y	z	
**FA3/4**	-1	5	34	15	15	12	0.207-1.973
FA6	-18	-12	18	20	15	18	0.536-1.915

References

1. Vanden Heuvel, J.P.; Kuslikis, B.I.; Peterson, R.E. Covalent Binding of Perflourinated Fatty Acids to Proteins in the Plasma, Liver and Testes of Rats. *Chemical-Biological Interactions* **1992**, *82*, 317–328.
2. Hirs, C.H.W.; Stein, W.H.; Moore, S. The Free Amino Acids of Human Blood Plasma. *Journal of Biological Chemistry* **1954**, *211*, 941–950.
3. Otter, D.E. Standardised Methods for Amino Acid Analysis of Food. *British Journal of Nutrition* **2012**, *108*, 230–237, doi:10.1017/S0007114512002486.
4. Muñoz, A.; Kral, R.; Schimmel, H. Quantification of Protein Calibrants by Amino Acid Analysis Using Isotope Dilution Mass Spectrometry. *Analytical Biochemistry* **2011**, *408*, 124–131, doi:10.1016/j.ab.2010.08.037.
5. Mustățea, G.; Ungureanu, E.L.; Iorga, E. Protein Acidic Hydrolysis for Amino Acids Analysis in Food - Progress over Time: A Short Review. *Journal of Hygienic Engineering and Design* **2019**, *26*, 81–87.
6. Moschet, C.; Anumol, T.; Lew, B.M.; Bennett, D.H.; Young, T.M. Household Dust as a Repository of Chemical Accumulation: New Insights from a Comprehensive High-Resolution Mass Spectrometric Study. *Environmental Science and Technology* **2018**, *52*, 2878–2887, doi:10.1021/acs.est.7b05767.
7. Barzen-Hanson, K.A.; Roberts, S.C.; Choyke, S.; Oetjen, K.; McAlees, A.; Riddell, N.; McCrindle, R.; Ferguson, P.L.; Higgins, C.P.; Field, J.A. Discovery of 40 Classes of Per- and Polyfluoroalkyl Substances in Historical Aqueous Film-Forming Foams (AFFFs) and AFFF-Impacted Groundwater. *Environmental Science and Technology* **2017**, *51*, 2047–2057, doi:10.1021/acs.est.6b05843.
8. Schymanski, E.L.; Jeon, J.; Gulde, R.; Fenner, K.; Ruff, M.; Singer, H.P.; Hollender, J. Identifying Small Molecules via High Resolution Mass Spectrometry: Communicating Confidence. *Environmental Science and Technology* **2014**, *48*, 2097–2098, doi:10.1021/es5002105.
9. Snow, A.W.; Giles, S.; Hinnant, K.M.; Farley, J.P.; Ananth, R. Quantification of Fluorine Content in AFFF Concentrates. **2017**.
10. Curry, S. Plasma Albumin as a Fatty Acid Carrier. *Advances in Molecular and Cell Biology* **2003**, *33*, 29–46.
11. Ackley, D.C.; Rockich, K.T.; Baker, T.R. *Optimazation of Drug Discovery: In Vitro Methods*; 2004; ISBN 1588293327.
12. Ng, C.A.; Hungerbuehler, K. Exploring the Use of Molecular Docking to Identify Bioaccumulative Perfluorinated Alkyl Acids (PFAAs). *Environmental Science and Technology* **2015**, *49*, 12306–12314, doi:10.1021/acs.est.5b03000.
13. Luo, Z.; Shi, X.; Hu, Q.; Zhao, B.; Huang, M. Structural Evidence of Perfluorooctane Sulfonate Transport by Human Serum Albumin. *Chemical Research in Toxicology* **2012**, *25*, 990–992, doi:10.1021/tx300112p.
14. Sander, T.; Freyss, J.; Von Korff, M.; Rufener, C. DataWarrior: An Open-Source Program for Chemistry Aware Data Visualization and Analysis. *Journal of Chemical Information and Modeling* **2015**, *55*, 460–473, doi:10.1021/ci500588j.
15. Wahl, J.; Freyss, J.; von Korff, M.; Sander, T. Accuracy Evaluation and Addition of Improved Dihedral Parameters for the MMFF94s. *Journal of Cheminformatics* **2019**, *11*, 1–10, doi:10.1186/s13321-019-0371-6.
16. Gunasekar, S.; Lee, J.; Soudry, D.; Srebro, N. Characterizing Implicit Bias in Terms of Optimization Geometry. *35th International Conference on Machine Learning, ICML 2018* **2018**, *4*, 2932–2955.
17. Hanwell, M.D.; Curtis, D.E.; Lonie, D.C.; Vandermeersch, T.; Zurek, E.; Hutchison, G.R. Avogadro: An Advanced Semantic Chemical Editor, Visualization, and Analysis Platform. *Advances in Mathematics* **2012**, *4*, doi:10.1016/j.aim.2014.05.019.
18. Pettersen, E.F.; Goddard, T.D.; Huang, C.C.; Couch, G.S.; Greenblatt, D.M.; Meng, E.C.; Ferrin, T.E. UCSF Chimera - A Visualization System for Exploratory Research and Analysis. *Journal of Computational Chemistry* **2004**, *25*, 1605–1612, doi:10.1002/jcc.20084.
19. Allendorf, F.; Berger, U.; Goss, K.U.; Ulrich, N. Partition Coefficients of Four Perfluoroalkyl Acid Alternatives between Bovine Serum Albumin (BSA) and Water in Comparison to Ten Classical Perfluoroalkyl Acids. *Environmental Science: Processes and Impacts* **2019**, *21*, 1852–1863, doi:10.1039/c9em00290a.
